# Gender differences in estimated excess mortality during the COVID-19 pandemic in Thailand

**DOI:** 10.1186/s12889-023-16828-9

**Published:** 2023-10-02

**Authors:** Wiraporn Pothisiri, Orawan Prasitsiriphon, Jutarat Apakupakul, Kritchavat Ploddi

**Affiliations:** 1 College of Population Studies, Chulalongkorn University, Bangkok, Thailand; 2 Division of Epidemiology, Department of Disease Control, Ministry of Public Health, Nontaburi, Thailand

**Keywords:** COVID-19, Excess death, SARIMA, Gender difference, Gender inequality

## Abstract

**Background:**

There is a limited body of research specifically examining gender inequality in excess mortality and its variations across age groups and geographical locations during the COVID-19 pandemic. This study aims to fill this gap by analyzing the patterns of gender inequality in excess all-cause mortality in Thailand during the COVID-19 pandemic.

**Methods:**

Data pertaining to all-cause deaths and population between January 1, 2010, and December 31, 2021, were obtained from Thailand’s Bureau of Registration Administration. A seasonal autoregressive integrated moving average (SARIMA) technique was used to estimate excess mortality during the pandemic between January 2020 to December 2021. Gender differential excess mortality was measured as the difference in age-standardized mortality rates between men and women.

**Results:**

Our SARIMA-based estimate of all-cause mortality in Thailand during the COVID-19 pandemic amounted to 1,032,921 deaths, with COVID-19-related fatalities surpassing official figures by 1.64 times. The analysis revealed fluctuating patterns of excess and deficit in all-cause mortality rates across different phases of the pandemic, as well as among various age groups and regions. In 2020, the most pronounced gender disparity in excess all-cause mortality emerged in April, with 4.28 additional female deaths per 100,000, whereas in 2021, the peak gender gap transpired in August, with 7.52 more male deaths per 100,000. Individuals in the 80 + age group exhibited the largest gender gap for most of the observed period. Gender differences in excess mortality were uniform across regions and over the period observed. Bangkok showed the highest gender disparity during the peak of the fourth wave, with 24.18 more male deaths per 100,000.

**Conclusion:**

The findings indicate an overall presence of gender inequality in excess mortality during the COVID-19 pandemic in Thailand, observed across age groups and regions. These findings highlight the need for further attention to be paid to gender disparities in mortality and call for targeted interventions to address these disparities.

**Supplementary Information:**

The online version contains supplementary material available at 10.1186/s12889-023-16828-9.

## Introduction

Estimating the true burden of the COVID-19 pandemic in terms of the number of lives lost is crucial for researchers and public health policymakers. Without sufficient and accurate data, such an endeavor is impossible, and any efforts to overcome the manifold pandemic-associated challenges would be implausible. Worldwide, there is a widespread belief that COVID-19-related deaths are underreported, with resource-poor countries being commonly assumed to have more unreported COVID-19 deaths, largely due to underdeveloped civil registration and vital statistics systems [1, 2]. However, increasing evidence suggests that many developed countries, including the US and several European nations, are also facing similar issues of undercounting COVID-19 deaths [3–5], signaling to other nations that they must pay greater attention to surveilling their COVID-19-related deaths.

Excess mortality—defined as the difference between the number of deaths during the pandemic and the number of deaths expected in normal years—has increasingly gained favor over official death statistics among epidemiologists and researchers in providing a more accurate assessment of the impact of COVID-19 on mortality [4–7]. Based on estimations of excess deaths, global deaths during the COVID-19 pandemic were calculated to be approximately 17.1–19.6 million between January 1, 2020, and December 31, 2021 [8, 9], about three times higher than the reported number of 5.9 million deaths. Additionally, there is emerging evidence for significant variations in excess mortality across sociodemographic subgroups, particularly regarding sex and geographical location [10–13].

More than two years into the pandemic, it has been well-documented that men have had greater disadvantages in terms of COVID-19 mortality according to several death reports and studies [14–17]. While this has yielded reasonable speculation that the pandemic will exacerbate existing gender inequalities through higher male mortality, findings from recent studies have suggested that it may be too early to draw definitive conclusions. For example, a study by Krieger et al. [18] has found no difference in the relative increase in excess mortality between men and women in the US during the early phase of COVID-19, even though the mortality rate was found to be higher for men. Meanwhile, a European-based study conducted by Nielsen et al. [16] showed that women had remarkably higher excess mortalities in some periods compared to men. A more recent study by Akter [19] revealed a reverse gender gap in mortality in 37 states across the US, shedding light on the geographical variation in gender inequality in terms of mortality during the pandemic.

A vast array of literature on the pandemic regarding its economic, health, social, and public policy consequences has consistently demonstrated that COVID-19 does not impact people uniformly across countries or even within the same country. A variety of contextual conditions and factors, such as population density, geographical features, land use patterns, demographic structures, urbanization levels, healthcare systems and their capacities, and public policies handling the pandemic, have all been reported as significant factors influencing COVID-19 deaths [19, 20]. However, evidence for how and to what extent these factors have affected mortality is mixed and inconclusive. For example, a study of several European countries found lower excess mortality in rural areas compared to peri-urban and urban areas due to lower population density and industrial land use, and limited social connectivity [21]. Other studies have reported inconsistent findings [20, 22], offering possible explanations involving the relatively greater socio-economic disadvantages of rural residents associated with limited healthcare access and resources [19, 20, 22]. There is also evidence pointing to marked gender inequalities during the pandemic with respect to healthcare access and utilization, with women often facing a higher mortality risk than men, leading to smaller or inverse gender gaps in mortality [17].

This study was motivated by the mixed evidence of gender inequality in mortality during the pandemic, coupled with the fact that very few studies have been conducted to specifically explore gender inequality using a measure of excess mortality and examine how it varies across COVID-19 periods, age groups and geographies. Among the studies available, all have focused on populations in developed nations. With the exception of Akter [19], these studies have only offered national-level estimates, neglecting the influence of geographical nuances on COVID-19 mortality rates.

Thailand, where the current study is based, provides a compelling setting for this research inquiry. Between January 2020 and December 2021, the official report showed that 21,700 deaths were assigned to COVID-19 [8]. However, Wang et al. [8] estimated that the provisional excess death count could be up to 42,200. Despite its relatively small number of deaths compared to other countries, Thailand was the first country in Southeast Asia to report a laboratory-confirmed COVID-19 case on January 17, 2020 [23] and among the first nations to impose a nationwide lockdown followed by curfews in April 2020, at which time the daily infected cases had peaked at 188, and the cumulative death toll had only reached 57—a relatively small number compared to other western nations [24]. As a result, the Thai government was highly praised by the international community for its effective COVID-19 response. Nonetheless, the country was hit by the second wave of COVID-19 during the December 2020–February 2021 period due to labor trafficking, shortly followed by the third wave between April and June 2021, with the average death tolls rising to 19 per day [25]. The fourth wave, taking place between July and December 2021, was seen as the worst hit by the pandemic up to that point, with the daily death toll reaching 312 lives lost per day [26].

The primary aim of this study was to examine and better understand the pattern of gender inequality in mortality during the COVID-19 pandemic in Thailand. To achieve this overarching aim, our study pursued three specific objectives. First, considering the possibility of underreported COVID-19 deaths based on the officially published data by the government, we intended to estimate the monthly excess all-cause mortality for both men and women from January 2020 to December 2021. To accomplish this, we employed the Box-Jenkins seasonal autoregressive integrated moving average (SARIMA) technique, a classical time-series model widely used in epidemiological research to account for temporal and seasonal changes in time-series data [27, 28].

Our second objective was to examine the differences in excess all-cause mortality between men and women, specifically focusing on how these differences varied over the two consecutive years investigated, as well as across different age groups and regions. As we recognized that factors such as the mutation of the COVID-19 virus and the implementation of public health measures could potentially influence the fluctuation of excess mortality patterns over time in conjunction with the existing evidence on regional disparities in mortality [29, 30], our study aimed to shed light on the dynamic nature of gender-related impacts during the COVID-19 pandemic. The age-disaggregated and region-disaggregated details will serve as key input for formulating policies and measures and the corresponding resource allocation for specific age groups and regions [31].

Finally, we investigated the differences between patterns of gender inequality in excess mortality derived from our model and those based on the reported COVID-19 death data by the official surveillance system of the Ministry of Public Health. This comparison emphasizes the significance of independent research in complementing and validating official sources, as it corroborates and strengthens the evidence pertaining to gender inequality in excess mortality.

## Data and methods

### Data

#### Mortality data

We obtained all-cause death counts—disaggregated by age, sex, province, and month between January 1, 2010, and December 31, 2021—through a written request submitted to the Bureau of Registration Administration (BORA) of Thailand’s Ministry of Interior (MOI). To adjust for potential delays in reporting deaths, the date of death and the date the administrative office was notified were also requested. Furthermore, the Department of Disease Control (DDC) in the Ministry of Public Health provided us with additional data on daily coronavirus-related deaths by age, sex, and province occurring between March 1, 2020 (the date of the first officially identified COVID-19 death in Thailand) and December 31, 2021.

#### Population data

Monthly population data by age, sex, and province were retrieved from the official website of the Population Registration Database, administered by BORA (https://stat.bora.dopa.go.th/stat/statnew/statMenu/newStat/home.php).

### Methods

#### Data preparation

The 1991 Civil Registration Law in Thailand stipulates that a person’s death must be reported to a government officer within 24 hours [32]. However, our preliminary review of the all-cause mortality data during a 12-year observation period revealed that the mode of reporting time was within 1 day after death, with an average of 2.81 days and a standard deviation (SD) of 37.81 days. The large SD value was mainly due to the wide distribution of data, with the maximum delay in reporting death of more than 4,300 days. Although the number of deaths incurred but not reported (IBNR) was relatively low, there was a potential delay of more than one month before these deaths were officially reported. To account for such delays, we adopted a chain ladder technique [33], in which the inflation factors by month and year, separated for men and women, were calculated and then used to estimate the total monthly all-cause deaths.

From January 1, 2010, to December 31, 2021, there were 5,634,224 observed deaths in Thailand. Due to invalid information on the death date, province, or sex, 3,663 deaths were excluded from the analysis. By adjusting for IBNR deaths, which accounted for approximately 2,058 deaths, the final number of observed deaths included in our analysis was 5,632,238.

To create a comparable metric, the daily COVID-19 mortality data from a DDC surveillance system were converted to monthly data. While we recognized the greater potential for reporting delays among COVID-19 deaths [34, 35], we were unable to account for such delays due to missing and invalid information on the date of death for approximately half of the total deaths. Between March 1, 2020, and December 31, 2021, the observed number of COVID-19 deaths was 21,698. By removing five cases without valid information on sex, the final observed number of COVID-19 deaths was 21,693.

To calculate monthly mortality rates, we needed to determine the person-months of exposure to the risk of dying. In this study, the mid-month population—calculated by averaging the initial and final population sizes at month $x$ and month $x$+1—was used as an estimate of person-months at risk. To minimize the confounding effect of age structure on mortality, which could lead to misinterpreting the results, particularly when making comparisons between men and women, we standardized age-specific monthly mortality rates using the direct method and population age structure from 2019. Additionally, to generate regional estimates, the province-level mortality data were aggregated to the regional level following the administrative classification of regions by the MOI.

### Statistical analysis

Monthly excess mortality is defined as the difference between the expected (baseline) number of deaths and the observed number of deaths taking place in a particular period of time [8]. The expected number of deaths by age, region, and gender was estimated based on the all-cause mortality patterns from 2010 to 2019 using the Box-Jenkins approach, also known as ARIMA model. The ARIMA ($$p,d,q)$$ model is a time-series model commonly employed in epidemiological research to capture the temporal dependence structure of a univariate time-series [27]. The letters $$p$$, $$d$$, and $$q$$ signify the order of autoregression, degree of difference, and order of moving average, respectively. The ARIMA model is considered appropriate when the data pattern is not repeated within a year. However, if the data pattern is seasonal, the extended version of ARIMA, called the seasonal ARIMA (SARIMA), is a more suitable model. The SARIMA model is specified as ($$p,d,q)\left(P,D,Q\right)\left[s\right]$$, in which the four additional hyper-parameters ($$\left(P,D,Q\right)\left[s\right]$$) represent the seasonal components, namely seasonal autoregression, seasonal integration, seasonal moving average, and seasonal period length, respectively. The literature has shown that SARIMA models have been proven and validated for capturing and estimating excess mortality during the COVID-19 pandemic [36–39].

Prior to the model fitting, we first examined the pattern of sex-specific all-cause mortality occurring between 2010 and 2019. The trends, seasonality, and stationary state of the data in the series were analyzed using time-series plots and the Augmented Dickey-Fuller (ADF) unit root test. If the ADF test indicated that the series of monthly mortality data was a non-stationary sequence (p ≥ 0.05), a transformation into a stationary time series was performed by taking a suitable difference to stabilize the variances. Table 1 shows the ADF test results for the data series for men and women and according to their ages and regions, indicating that all data series were in a stationary state with *p*-values of less than 0.05.

**Table 1:** Results of the augmented Dickey-Fuller test for men and women

	Men			Women	
	Dickey-Fuller	*p*-value		Dickey-Fuller	*p-*value
***Total***	-5.27	0.01		-4.95	0.01
***Age group***					
0–14	-4.04	0.01		-4.72	0.01
15–34	-5.99	0.01		-3.52	0.04
35–59	-4.46	0.01		-3.54	0.04
60–69	-4.17	0.01		-4.21	0.01
70–79	-5.14	0.01		-4.90	0.01
80+	-5.81	0.01		-5.84	0.01
***Region***					
Bangkok	-4.50	0.01		-3.92	0.02
Central	-4.65	0.01		-4.74	0.01
North	-5.61	0.01		-5.61	0.01
South	-5.70	0.01		-5.55	0.01
Northeast	-5.42	0.01		-4.96	0.01

**Fig. 1:**
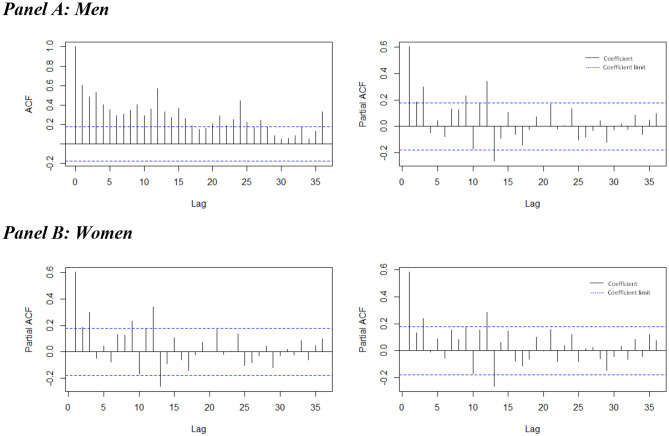
The ACF and PACF plots for the data series for men and women (d = 0, D = 0)

The “auto.arima( )” command provided in the “Forecast” package in R program (version 4.0.3) was then used to generate the autocorrelation function (ACF) and partial autocorrelation function (PACF) diagrams, and later to identify the best-fitted SARIMA models. The ACF and PACF plots based on the original data series were visually and supplementarily examined to confirm the stationary state and suggest possible orders of the autoregressive and moving average components of the SARIMA models. Figure 1 exhibits the examples of the ACF and PACF plots, with 95% significance boundaries shown in dotted lines, for the original data series for men and women (d = 0, D = 0). The plots for the original data series by age groups and regions for men and women can be found in supplementary information.

In the process of building the SARIMA model, the “auto.arima” function performs an iterative procedure to estimate several SARIMA models with different orders of SARIMA(p,d,q)(P,D,Q)[s], and automatically chose the optimal model based on the Akaike information criterion (AIC) [40]. To validate the predictability of the fitted model, as suggested by previous studies [41, 42], the Ljung-Box Q statistics were performed again for lags 1, 5, and 20 to ensure that the residuals exhibited no autocorrelation for a fixed number of lags and that the fitted model captured all trends in the data series [43–45]. All SARIMA models obtained by the auto.arima command were confirmed by the Ljung-Box Q statistics, except for the women residing in the Southern region. For this particular data series, the originally identified model was SARIMA (0,1,2)(2,0,0) [12], with a Ljung-Box Q value of 38.52 and a corresponding *p*-value of 0.01. As such, the model was manually adjusted by using a seasonal differencing order D of 1 to SARIMA (0,1,2)(2,1,0) [12] to improve the fit, which yielded a Ljung-Box Q value of 20.75 and a corresponding *p*-value of 0.44.

All data analyses were performed using STATA (version 17) and were separated by sex, age group (i.e., 0–14, 15–34, 35–59, 60–69, 70–79, and 80 years or over), and five regions (Bangkok, Central, North, South, and Northeast). The number of excess deaths was reported in both absolute and relative (to 100,000 population) terms. The predicted numbers of deaths and death rates were presented with 95% confidence intervals (CIs). For death counts, the 95% CIs were calculated as${\text{N}}_{1}\pm 1.96\text{*}{\text{S}\text{E}}_{1}$ with N_1_ and SE_1_ representing the monthly death count and the standard error, respectively, in which SE_1_ was calculated using a Poisson distribution as $\sqrt{{\text{N}}_{1}}$ [46, 47]. For age-standardized death rates, the 95% CIs were calculated as${\text{N}}_{2}\pm 1.96\text{*}{\text{S}\text{E}}_{2}$, with N_2_ and SE_2_ representing the monthly age-standardized death rate and standard error, respectively, in which SE_2_ was calculated based on a binomial proportion as ${\text{N}}_{2}/\sqrt{{\text{N}}_{1}}$, taking into account the variability in death counts and rates, as described by Keyfitz [48].

## Results

This section is structured as follows. First, we introduce the observed monthly all-cause mortality between 2010 and 2019 by gender, age, and region. Next, we present the results from fitting these data into the SARIMA models to derive the expected all-cause mortality during the COVID-19 pandemic for men and women, overall and by age and region. Based on the modeling results, we then examine the gender differences in excess mortality during the COVID-19 pandemic for the overall population and with respect to age groups and regions. Finally, we compare the results from the analyses of gender differences in excess all-cause mortality, including the overall differences and the variations by age and region, to those derived from the official COVID-19 death counts reported by the Thai government.

### Trends in observed monthly mortality between 2010 and 2019

**Fig. 2:**
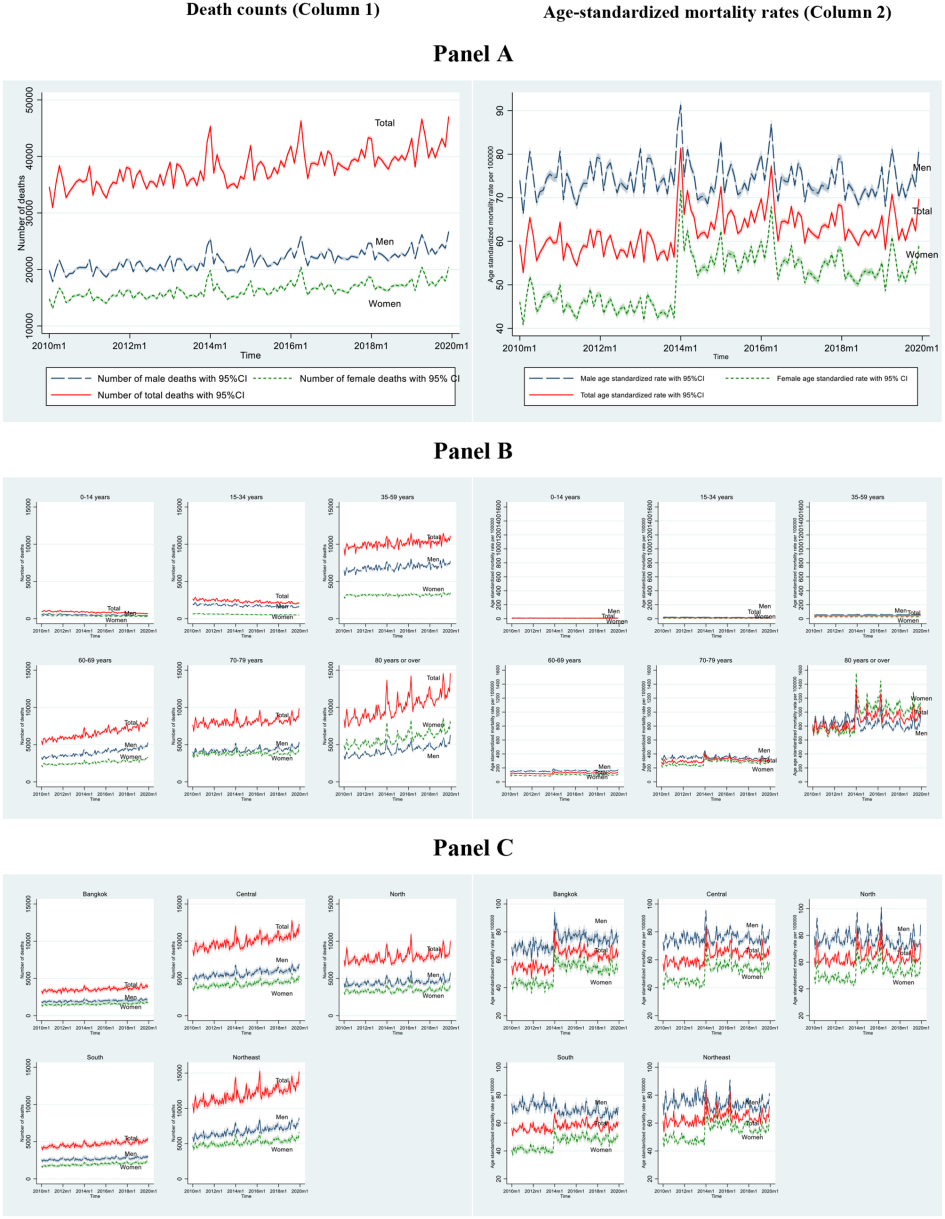
Observed death counts and age-standardized mortality rates (per 100,000 person-months), January 2010-December 2019, Thailand

There were 4,573,706 observed all-cause deaths between January 1, 2010, and December 31, 2019, of which 56.93% were men and 43.07% were women. The average monthly number of deaths for the total, male, and female populations during these years were 38,031, 21,650, and 16,381, respectively. The time-series data, as displayed in Fig. 2, Panel A, Column 1, exhibits a clear upward trend in overall mortality, reflecting the rapid population aging taking place in Thailand recently, with the share of the Thai population aged 60 years and older substantially increasing from 12.94% to 19.22% between 2010 and 2020 [49]. A similar trend was also observed for both the male and female populations. Furthermore, the results indicate a gender gap in mortality, primarily advantaging women, overall and across age groups and regions. In all age groups except for those over 80 years old, the mortality counts were higher for men than for women. The gender gap was largest in the 35-59-year-old group and smallest in the 0-14-year-old group (Fig. 2, Panel B, Column 1). These gender differences in mortality also varied by region, with the Northeast region exhibiting not only the highest mortality levels in both sexes but also the largest gender difference compared to other regions (Fig. 2, Panel C, Column 1).

In terms of age-standardized mortality rates, the average monthly death rates from 2010 to 2019 were 62.34 per 100,000 for the overall population, 74.27 per 100,000 for men, and 51.42 per 100,000 for women. In contrast to our previous observation in absolute terms, the results in relative terms demonstrate inconsistent patterns between men and women. The mortality rates for men varied narrowly between 70 and 80 per 100,000 over the period covered, while a sharp rise in mortality rates was observed for women in early 2014, after which the rates remained relatively stable (Fig. 2, Panel A, Column 2). Combined with the absence of an increasing trend in the mortality rates for men, this resulted in notably smaller gender differences between 2014 and 2019.

Gender differences in mortality rates over this period were further examined regarding age groups and regions. In terms of age group, mortality rates were generally lower for women across all age groups except for the 80 + group (Fig. 2, Panel B, Column 2). In the population aged 60 years and older, two distinguished patterns were observed between 2014 and 2019: a smaller gender gap, which was prevalent among the population aged 60–79 years, and a reverse gender gap with a higher level of mortality among women in the 80 + age group.

Unlike age groups, the pattern of gender differences in mortality rates over time varied only slightly across regions. An increasing level of mortality over time was observed only in men living in Bangkok, while similar patterns were prevalent among women in all regions. This resulted in relatively smaller gender differences for all regions compared to Bangkok.

### Model fitting and expected monthly mortality between 2010 and 2019

Table 2 presents the parameters of the fitted SARIMA models based on the observed monthly mortality time-series data between January 2010 to December 2019. For all the fitted models, the *p-*values in the Ljung-Box Q test for residuals were greater than 0.05, indicating that the fitted SARIMA models contained all the trends in the data series reasonably well. These models could thus be used to forecast the expected all-cause mortality rates between January 2020 and December 2021.

**Table 2:** Parameters of the fitted Seasonal Autoregressive Integrated Moving Average (SARIMA) models ((p, d, q) (P, D, Q) [s]) and selection criteria values for the time-series data from January 2010 to December 2019 for men and women and by age and region

	SARIMA	Model fit statistics		Ljung-Box Q					
				Degree of freedom = 1		Degree of freedom = 5		Degree of freedom = 20	
	(p, d, q)(P, D, Q)[s]	AIC		Statistics	*p-*value	Statistics	*p-*value	Statistics	*p-*value
**Men**									
***Total***	SARIMA (1,0,0)(2,1,0) [12]	1,751		0.20	0.65	5.76	0.33	22.55	0.31
***Age group***									
0–14	SARIMA (0,1,1)(0,0,2) [12]	1,173		2.27	0.13	5.99	0.31	19.81	0.47
15–34	SARIMA (0,0,2)(1,1,1) [12]	1,193		0.08	0.78	1.28	0.94	17.35	0.63
35–59	SARIMA (2,0,2)(2,1,0) [12]	1,452		0.03	0.86	3.13	0.68	20.38	0.43
60–69	SARIMA (1,0,0)(2,1,0) [12]	1,380		0.38	0.54	7.79	0.17	23.59	0.26
70–79	SARIMA (1,1,1)(2,0,0) [12]	1,629		0.15	0.70	7.81	0.17	21.45	0.37
80+	SARIMA (2,1,1)(2,0,0) [12]	1,708		0.07	0.79	1.60	0.90	9.43	0.98
***Region***									
Bangkok	SARIMA (1,1,1)(2,0,0) [12]	1,396		0.21	0.65	5.75	0.33	17.14	0.64
Central	SARIMA (1,1,1)(2,0,0) [12]	1,669		0.92	0.34	6.25	0.28	17.39	0.63
North	SARIMA (1,0,0)(2,1,0) [12]	1,488		0.07	0.79	0.89	0.97	8.88	0.98
South	SARIMA (1,0,0)(0,1,1) [12]	1,279		0.06	0.81	0.73	0.98	10.37	0.96
Northeast	SARIMA (1,0,0)(2,1,0) [12]	1,563		0.09	0.76	1.92	0.86	23.58	0.26
**Women**									
***Total***	SARIMA (1,1,1)(2,0,0) [12]	1,937		0.27	0.60	4.72	0.45	15.59	0.74
***Age group***									
0–14	SARIMA (2,1,1)(2,0,0) [12]	1,136		0.03	0.87	0.76	0.98	15.39	0.75
15–34	SARIMA (0,1,1)(2,0,0) [12]	1,155		0.03	0.86	6.56	0.26	17.30	0.63
35–59	SARIMA (1,0,0)(2,1,0) [12]	1,302		0.06	0.80	2.30	0.81	15.21	0.76
60–69	SARIMA (1,1,1)(0,0,2) [12]	1,492		0.16	0.69	1.87	0.87	17.77	0.60
70–79	SARIMA (1,0,0)(2,0,0) [12]	1,614		0.34	0.56	2.78	0.73	12.32	0.90
80+	SARIMA (2,1,1)(2,0,0) [12]	1,793		0.09	0.76	1.34	0.93	10.42	0.96
***Region***									
Bangkok	SARIMA (5,1,1)(2,0,0) [12]	1,400		0.11	0.75	0.76	0.98	10.92	0.95
Central	SARIMA (1,1,1)(2,0,0) [12]	1,654		0.01	0.94	4.30	0.51	13.19	0.87
North	SARIMA (1,1,1)(2,0,0) [12]	1,659		0.33	0.57	3.26	0.66	10.17	0.97
South	SARIMA (0,1,2)(2,1,0) [12]	1,243		0.00	0.99	0.63	0.99	20.75	0.41
Northeast	SARIMA (1,1,1)(2,0,0) [12]	1,716		0.16	0.69	1.92	0.86	9.45	0.98

Note: The letter *p* represents the order of autoregression, *d* represents the degree of differencing, and *q* represents the order of moving average. The letters *P*, *D*, and *Q* represent the parameters for seasonal autoregression, seasonal integration, and seasonal moving average, respectively. The letter *s* denotes the length of the seasonal period. AIC stands for Akaike information criterion.

Table 3 presents the expected death counts for 2020 and 2021 individually and in both years combined. In the year January 1, 2020, and December 31, 2020, the total expected death counts were 514,261 (95% CI: 469,953–558,568), with 56.81% being men and 43.19% women. For 2021, the total expected deaths were 518,660 (95% CI: 467,135–570,185), of which 56.58% were men and 43.42% were women. Combining the two years resulted in a total expected death count of 1,032,921 (95% CI: 937,089–1,128,753), the male-to-female ratio for the two years combined was 1.31:1.

**Table 3:** All-cause excess mortality counts, Thailand, 2020–2021

	Observed deaths	Expected deaths			Excess deaths	
		Estimated counts	95% CI		Estimated counts	95% CI
**Total**	1,068,531	1,032,921	(937,089–1,128,753)		35,610	(35,240–35,980)
***2020***						
All	501,451	514,261	(469,953–558,568)		–12,810	(–13,032 to − 12,588)
Men	285,536	292,132	(270,984–313,279)		–6,596	(–6,755 to − 6,437)
Women	215,915	222,129	(198,970–245,289)		–6,214	(–6,369 to − 6,059)
***2021***						
All	567,080	518,660	(467,135–570,185)		48,420	(47,989–48,851)
Men	322,404	293,467	(270,097–316,836)		28,937	(28,604–29,270)
Women	244,676	225,193	(197,038–253,349)		19,483	(19,209–19,757)

Note: 95% CI stands for 95% confidence interval.

### Excess all-cause mortality during the COVID-19 pandemic

**Fig. 3:**
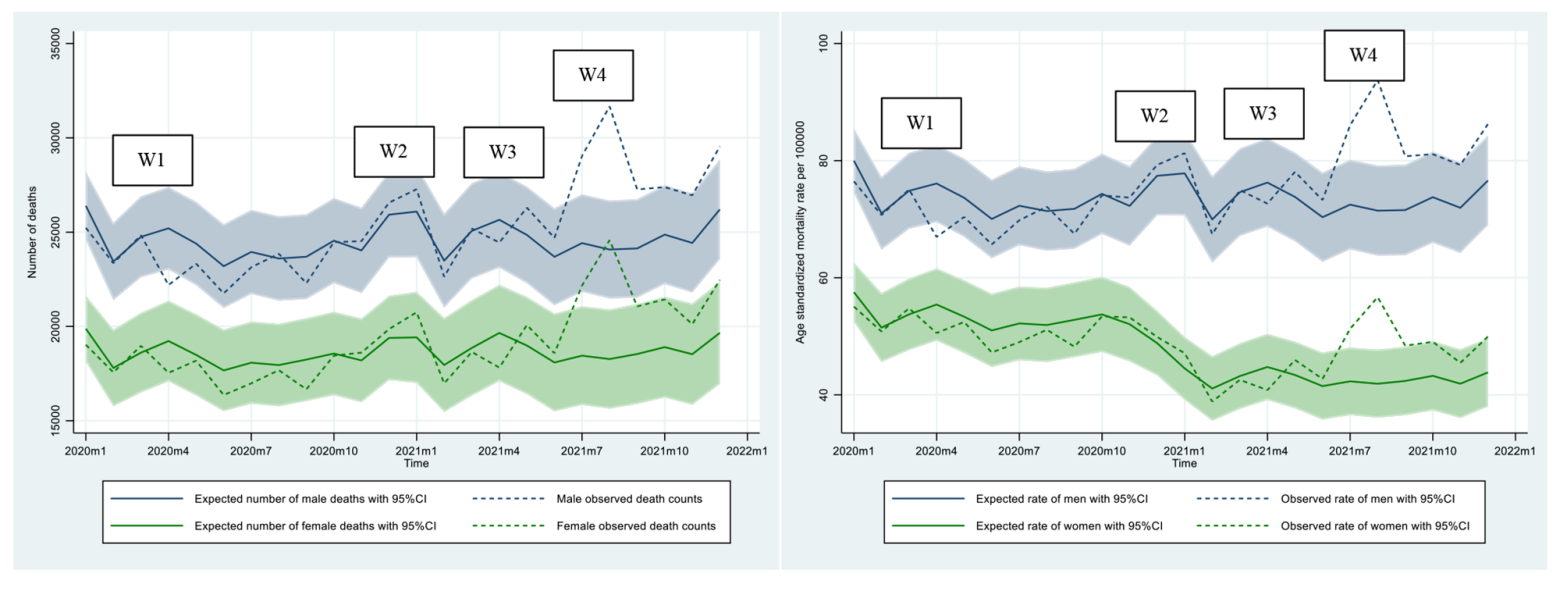
Expected and observed death counts and age-standardized rates (per 100,000 person-months) for men and women, January 2020-December 2021, Thailand Note: W1, W2, W3, and W4 denote the first (March-April 2020), second (December 2020-February 2021), third (April-May 2021), and fourth (July-December 2021) waves of the COVID-19 pandemic, respectively.

Figure 3 illustrates the plots of the expected all-cause mortality over time for men and women against the observed all-cause mortality on a monthly basis. The solid lines denote the expected death counts, while the shaded areas represent the associated 95% CIs. The dashed lines depict the corresponding observed death counts. Excess (or deficit) mortality is represented by a gap between the dashed and the solid lines.

The results show variations in excess (i.e., expected deaths < observed deaths) and deficit (i.e., expected deaths > observed deaths) mortality in both men and women during the pandemic, with the pattern varying substantially over the observed period. In 2020, mortality deficits largely manifested in the first three quarters of the year, while the mortality excesses emerged in an obvious way in the final quarter, which was apparent for both men and women. As shown in Table 3, the estimated all-cause excess death counts were negative at − 6,596 (95% CI: − 6,755 to − 6,437) for men, − 6,214 (95% CI: − 6,369 to − 6,059) for women, and − 12,810 (95% CI: − 13,032 to − 12,588) for both sexes combined. The male-to-female ratio for all-cause deficit deaths in 2020 was 0.94:1.

In stark contrast, mortality deficits in 2021 appeared sporadically in the first half of the year from February to May, followed by the predominance of mortality excesses throughout the rest of the year. This pattern was similar for both sexes. For 2021, the all-cause excess deaths estimated for men, women, and both sexes combined were 28,937 (95% CI: 28,604–29,270), 19,483 (95% CI: 19,209–19,757), and 48,420 (95% CI: 47,989–48,851), respectively. The corresponding male-to-female ratio was 1.49:1.

The results also demonstrate that the variation of mortality excesses and deficits over time in relative terms was, by and large, consistent with that measured in absolute terms for both men and women. However, there was a decreasing trend in death rates for women that occurred between September 2020 and January 2021.

### Gender differences in excess all-cause mortality

**Fig. 4:**
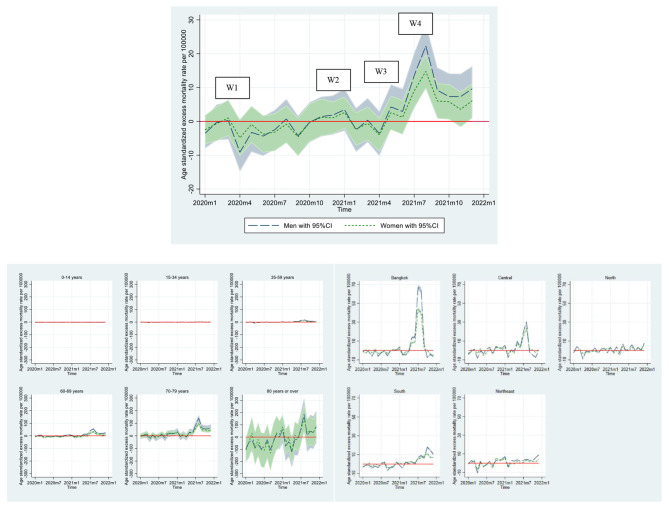
Age-standardized excess mortality rate per 100,000 person-months for men and women, by age group and region, January 2020-December 2021, Thailand Note: W1, W2, W3, and W4 denote the first (March-April 2020), second (December 2020-February 2021), third (April-May 2021), and fourth (July-December 2021) waves of the COVID-19 pandemic, respectively.

**Fig. 5:**
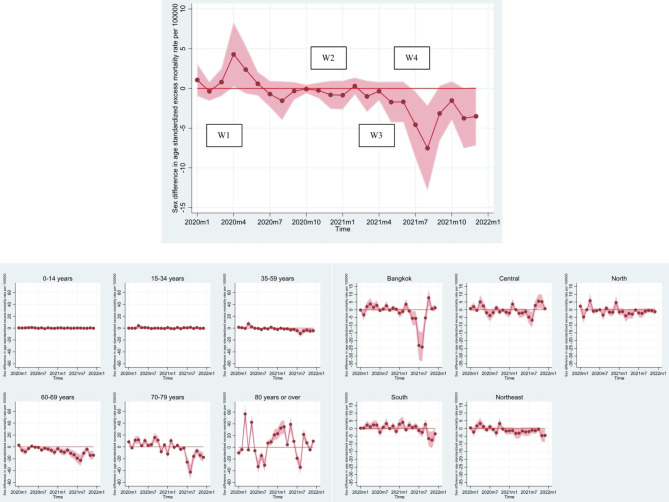
Gender differences in age-standardized excess mortality rate per 100,000 person-months by age group and region, January 2020-December 2021, Thailand Note: W1, W2, W3, and W4 denote the first (March-April 2020), second (December 2020-February 2021), third (April-May 2021), and fourth (July-December 2021) waves of the COVID-19 pandemic, respectively.

To examine gender differences in excess mortality for the overall population, as well as for age groups and regions, we focused on age-standardized mortality rates to minimize the potential confounding effects of age structure. The difference between the expected and observed mortality rates is presented in Fig. 4, with a positive value indicating excess mortality and a negative value representing deficit mortality. We computed the magnitude of these gender differences, presented in Fig. 5, by deducting the excess mortality rate for men from that of women, with a positive value showing a higher excess mortality rate.

As illustrated in Fig. 4, the results reveal that the excess mortality in men and women did not substantially differ overall. However, the two periods witnessed remarkable gender differences in excess mortality. The first period was April 2020, which coincided with the peak of the first wave of the COVID-19 outbreak. Notably, the excess mortality rates were negative for both men and women, with negative excess mortality rates being distinctively higher in men than women. The corresponding difference in excess mortality rates, as shown in Fig. 5, was 4.28 per 100,000 (= − 4.86 per 100,000 – − 9.13 per 100,000), suggesting that there were approximately four fewer excess deaths per 100,000 men than women. Another noteworthy phenomenon was observed in August 2021, which corresponded with the fourth wave of COVID-19. Men had a markedly higher excess mortality rate compared to women at − 7.52 per 100,000 (= 14.77 per 100,000 – 22.29 per 100,000), indicating that there were approximately eight more excess male deaths per 100,000.

### Gender differences in excess all-cause mortality by age groups

Figures 4 and 5 further show that gender differences in excess mortality increased with age, particularly for older age groups. In 2020, in the young and middle-aged populations, there were virtually no gender differences in excess mortality, even during the previously identified peak period in April. A somewhat similar trend is observed for 2021, with the exception of the middle-aged group aged 35–59 years during the second peak period in August 2021, where men exhibited higher excess mortality than women.

However, in the older age groups, gender differences in excess mortality were clearly observed and had contrasting patterns. In the 60–69 age group, gender differences in excess mortality were relatively modest compared to those in the 70–79 and 80 + age groups, with male showing higher excess mortality rates during most of the COVID-19 pandemic period. The corresponding gender differences in excess mortality, as shown in Fig. 5, ranged from − 22.76 per 100,000 to 2.83 per 100,000, with a remarkably higher male excess mortality of 22.76 per 100,000 during the fourth wave of COVID-19 in 2021.

Conversely, the middle-old and oldest-old age groups exhibited significantly wider gender differences in excess mortality, especially for the oldest old group. In the 70–79 age group, a noticeable difference from the youngest age group was observed: females experienced greater excess mortality than males for most of the COVID-19 pandemic period before the second peak in August 2021. Afterward, the pattern reversed, with the largest gender gap observed in the year 2021. The 80 + age group displayed the most diverse patterns of gender differences in excess mortality rates throughout the COVID-19 period, with the largest gender gap in excess mortality observed for the two years at 56.75 more deaths per 100,000.

### Gender differences in excess all-cause mortality by regions

In terms of region, Figs. 4 and 5 demonstrate modest variations in gender differences in excess mortality over time across regions. In 2020, all regions shared similar patterns of gender differences over time, with a slight variation in the range between − 4.68 and 100,000 and 5.85 per 100,000. However, these trends differed in 2021 where Bangkok experienced the largest gender differences in excess mortality (at − 24.18 per 100,000) during the peak of the fourth wave, followed by the Central region (at − 6.73 per 100,000). After this peak, men in the Southern and Northeastern regions showed higher excess mortality than women, while the opposite occurred in Bangkok and the Central region.

### Comparison of gender differences between predicted excess all-cause mortality and official COVID-19 death data

**Fig. 6:**
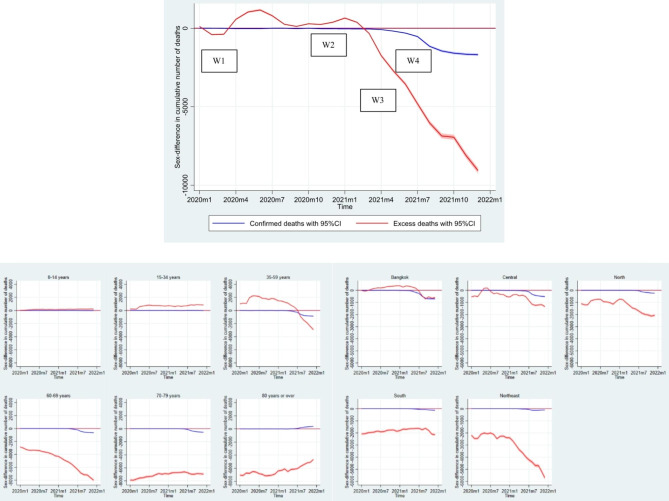
Gender differences (women-men) in officially reported COVID-19 deaths and predicted excess mortality counts, January 2020-December 2021, Thailand Note: W1, W2, W3, and W4 denote the first (March-April 2020), second (December 2020-February 2021), third (April-May 2021), and fourth (July-December 2021) waves of the COVID-19 pandemic, respectively.

Finally, we further investigated the gender differences in COVID-19 deaths that were registered in the official surveillance system of the Ministry of Public Health. Here, we sought to understand whether the gender differences in excess mortality we observed aligned with those that were officially reported. Figure 6 reveals the large discrepancy in the gender differences between registered COVID-19 deaths and estimated excess deaths, particularly in 2021, when the gap widened substantially over a few months. Upon closer examination of the data according to age group, a more intricate pattern of variation in these discrepancies over time emerged, with fewer excess deaths observed in comparison to registered COVID-19 deaths among older age groups, while the opposite trend was observed among the young and middle-aged groups. We also observed geographical variations, with most regions generally showing smaller gender differences in excess mortality over time compared to the registered COVID-19 deaths, except for Bangkok. In Bangkok, the gender differences in excess mortality over time were generally higher prior to the fourth wave of COVID-19, after which the gap between the gender differences in excess mortality and officially reported deaths decreased and then reversed towards the end of 2021.

## Discussion

Our study adds to the body of literature concerning the impact of the COVID-19 pandemic in terms of excess mortality and the associated gender differences over time in the developing context. Based on a ten-year historical mortality dataset, the all-cause mortality count carried out in Thailand between January 1, 2020, and December 31, 2021, was reported as 1,032,921 (95% CI: 937,089–1,128,753). Using the SARIMA model, our analysis suggests that 35,610 (95% CI: 35,240–35,980) people died due to COVID-19 in Thailand within the same period, which is 1.64 times higher than the official statistics. While this ratio provides evidence that COVID-19 deaths have been undercounted in Thailand, its magnitude is relatively small compared to that of other developing countries in the region and beyond [8].

Our study covered a period of two consecutive years during the COVID-19 pandemic and allowed us to analyze its impacts, which were driven by multiple factors, including mutations of the COVID-19 virus, sociodemographic status, and public health and social measures. The results of our study reveal mixed patterns of excesses and deficits in all-cause mortality throughout this period. During the first wave of the COVID-19 outbreak between March and April 2020, Thailand experienced deficits in mortality, which remained apparent until October 2020, prior to the second wave of the pandemic. After October, labor trafficking from neighboring countries [24, 50] resulted in a significant increase in excess mortality until January 2021. This phenomenon of mortality deficits followed by excesses during the first and second waves of the pandemic has been similarly reported in Central and Eastern European countries [51]. In 2021, the situation changed, with a marginal mortality deficit in the first two quarters followed by a mortality excess that remained throughout the year due to the outbreaks of the Alpha and Delta variants of the virus.

Our findings show that variations in excess mortality over the period examined were similar for both men and women and that there were no significant differences between genders. While previous studies have shown that men were more likely to die during the COVID-19 pandemic [14–17], our findings reveal that this was not necessarily the case in Thailand. During the first wave of the pandemic in April 2020, Thai men exhibited a remarkably greater deficit in excess mortality than women. A possible explanation could be the strict enforcement of and public obedience to the Thai government’s measures in response to the country’s first encounters with COVID-19. These measures, including lockdowns, social distancing, and travel restrictions, lowered transport mobility and, thus, the risk of road accidents, which are more likely to involve men than women [51, 52]. Prior to the outbreak of COVID-19, an average of 20,000 Thai people died from road accidents each year [53], with the highest fatalities taking place in April and during the new year holidays. Owing to the government’s measures during the first wave, road accidents claimed 770 lives in April, according to Road Accidental Statistics in Thailand [54], which is half of the corresponding figure from 2019 according to Statistics of Deaths and Injuries in Thailand [55]. After the first wave of COVID-19, however, the previously strict public safety measures were gradually lifted due to their negative impacts on economic activities and people’s lives. As a result, excess mortality among men increased once again during the fourth wave of the pandemic.

Our results confirm that gender differences in excess mortality were positively associated with age, aligning with the existing literature [12, 56]. In line with Akter’s study [19], we found that gender differences in excess mortality were more prominent in older age groups compared with younger ones. Within the older age groups, our study further demonstrates that the pattern and extent of gender differences over time varied substantially by age. Gender differences in excess mortality were largest among those in the 80 + age group during the first wave of COVID-19, with women exhibiting higher rates of excess mortality than men.

Gender differences in excess mortality during COVID-19 were also found to be geographically uneven, which is consistent with previous studies [19, 20, 22]. Our results further showed varying patterns of gender differences in excess mortality over time across different regions in Thailand. The largest gender difference in excess mortality was observed in Bangkok during the peak of the fourth wave. While population density and other contextual factors, such as the demographic structure and community contagion level, which was influenced by people’s daily movements, might explain why Bangkok had the highest number of infectious cases and level of mortality [57–59], it remains unclear how these factors superseded other relieving factors, such as health capacity and resources, which are most readily available in Bangkok. One possible explanation is that the demand for COVID-19 treatments overwhelmed the supply of Bangkok’s healthcare systems, thereby limiting access to healthcare. Notably, what caused the gender difference in excess mortality to vary across regions is still not fully understood and warrants further causal studies.

There were several limitations to this study that are worth mentioning. First, it excluded the underlying gender difference in all-cause mortality, which could affect the gender gap in excess mortality. Therefore, the results should be interpreted with caution. Second, although it is widely believed that a significant portion of the excess mortality during the pandemic can be attributed to COVID-19, we lacked sufficient empirical evidence to draw a definitive conclusion. We suggest that future studies examine other possible factors that may contribute to or impede excess mortality.

## Conclusion

Based on a ten-year historical mortality dataset, our study reveals several key findings regarding gender inequality in excess all-cause mortality during the COVID-19 pandemic in Thailand. First, there was a notable difference in excess mortality between Thai men and women. In the initial period of the pandemic, men experienced a greater deficit in excess all-cause mortality compared to women. However, in subsequent periods, men exhibited a larger excess in all-cause mortality. These differences could be attributed to the stronger enforcement of government measures and higher public compliance at the beginning of the pandemic. Second, we found that gender differences in excess mortality, expressed as differences in standardized age-specific mortality rates, increased with age, with more pronounced differences in the older age group. Finally, we observed variations in the gender-differential excess mortality over time across different regions. These findings contribute to the existing literature on the impact of the COVID-19 pandemic, specifically in terms of gender-differential excess mortality, within the context of a developing country. Moreover, they emphasize the importance of conducting further studies to investigate gender disparities in mortality and call for targeted interventions to address these disparities.

### Electronic supplementary material

Below is the link to the electronic supplementary material.

Supplementary Material 1

## Data Availability

The data used to support the findings of this study were obtained from the Bureau of Registration Administration (BORA) under Thailand’s Ministry of Interior (MOI) and the Department of Disease Control (DDC) under the Ministry of Public Health (MOH). These data were obtained with permission for use only in the current study and are not publicly available. If you have any further inquiries, please contact Dr. Orawan Prasitsiriphon at orawan.pr@chula.ac.th.
