# Supplementary material for: Gender differences in estimated excess mortality during the COVID-19 pandemic in Thailand
Source: BMC Public Health. 2023 Oct 2;23:1900. doi: 10.1186/s12889-023-16828-9 (PMC10544589; doi:10.1186/s12889-023-16828-9)
Supplement: Supplementary file 1 — Supplementary Material 1 [file 12889_2023_16828_MOESM1_ESM.docx]

**Additional file 1: Figure S1.**

Data pertaining to monthly population data between January 1, 2010, and December 31, were obtained from Thailand’s Bureau of Registration Administration. Data was plotted for the ACF and PACF as shown in Figure S1, before we identified the final model by using the auto.arima command in R programs.

**Figure S1.** The ACF and PACF plots for the data series by gender, age groups, and regions.

| **Test** | **ACF** | **PACF** |
| --- | --- | --- |
| **Men** | | |
| ***Total*** | 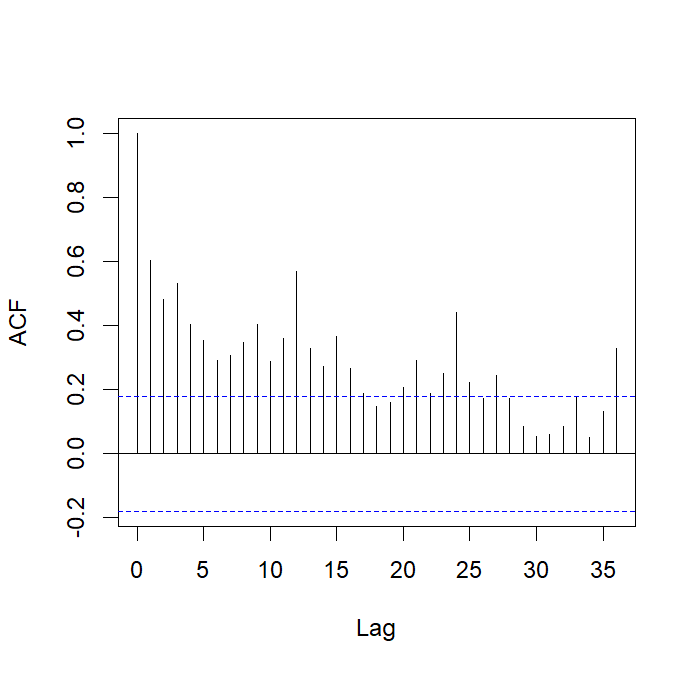 | **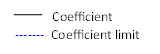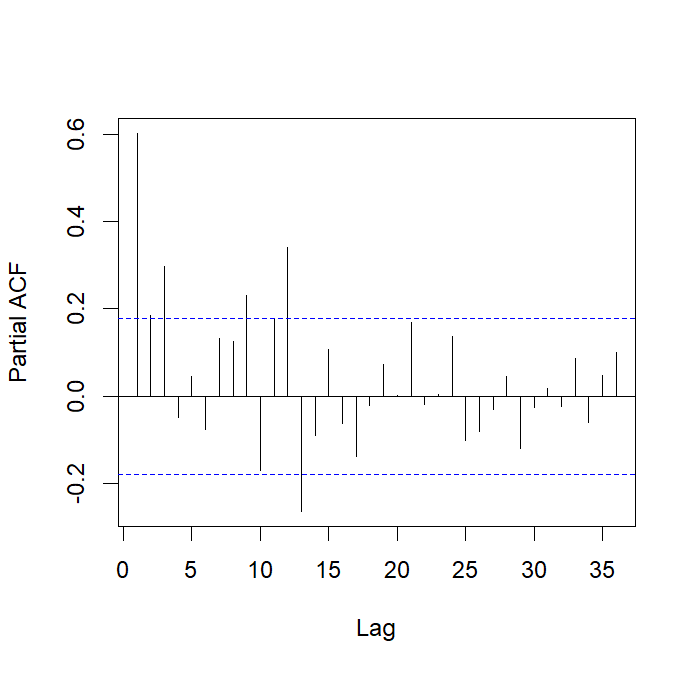** |
| ***Age group*** |  |  |
| 0–14 | 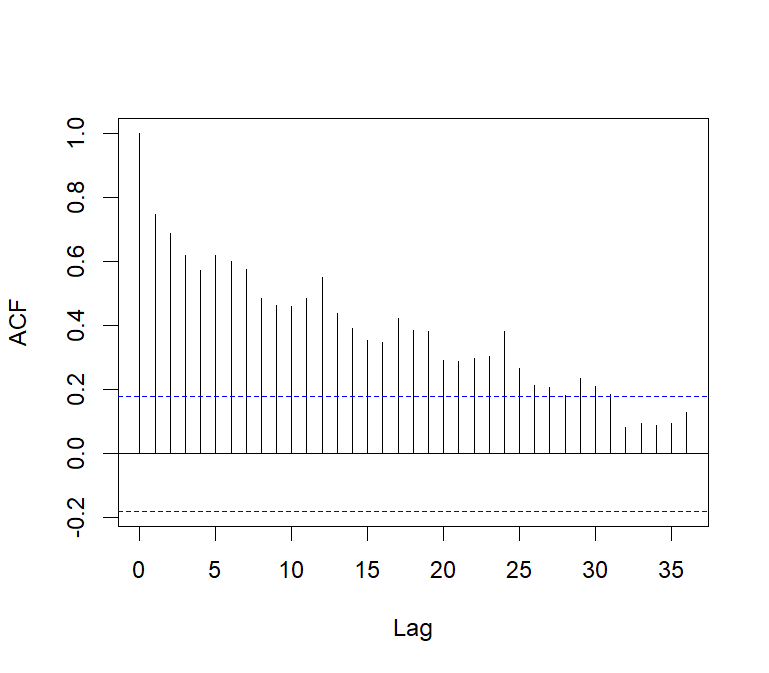 | **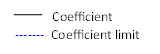**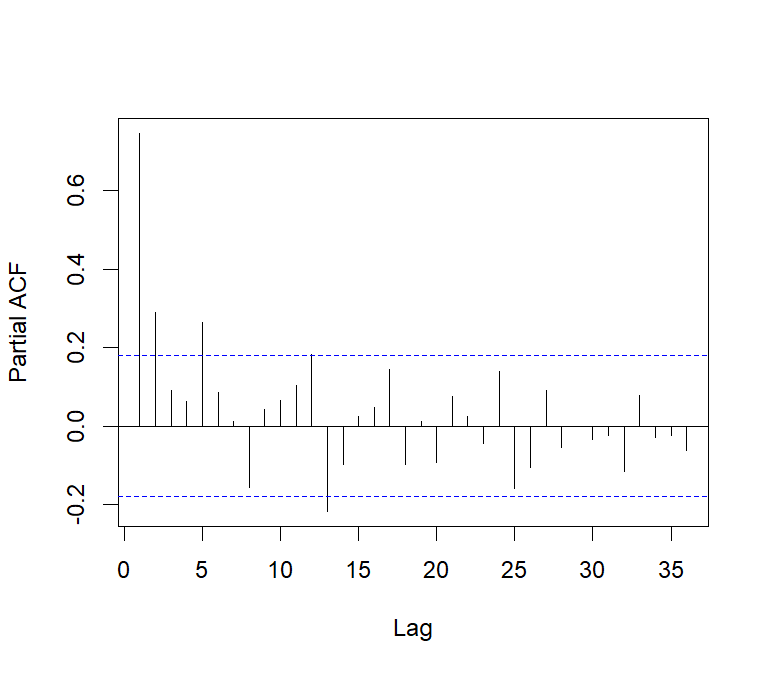 |
| 15–34 | 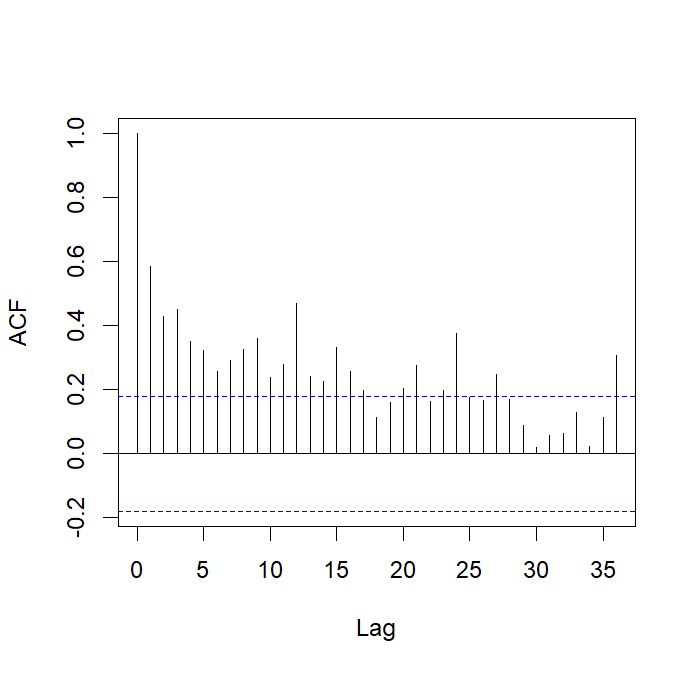 | **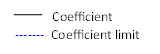**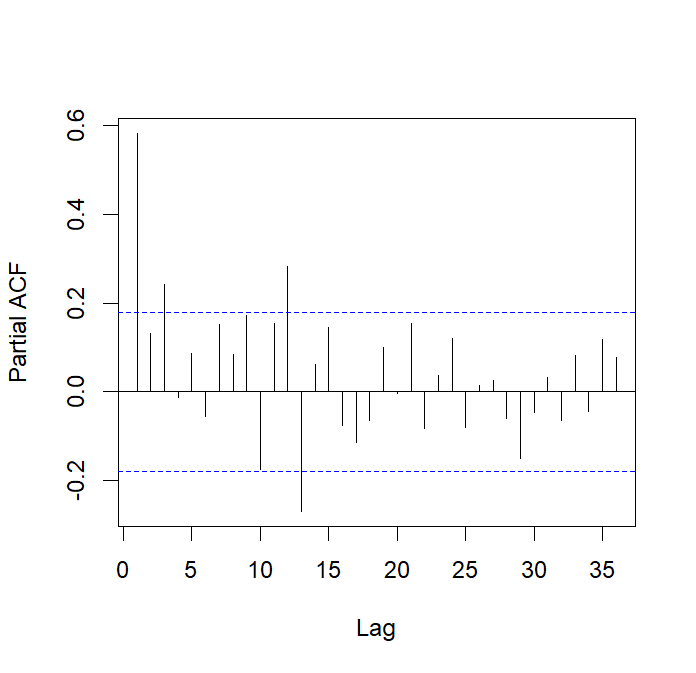 |
| 35–59 | 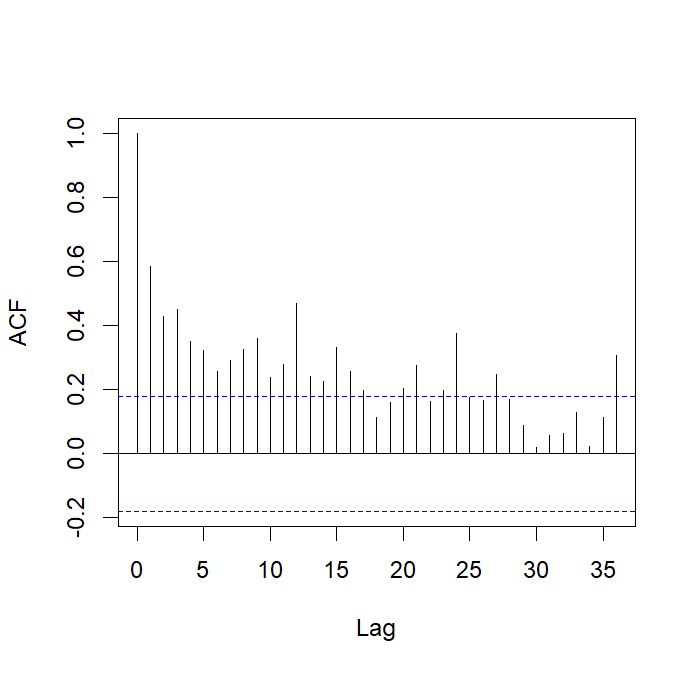 | **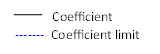**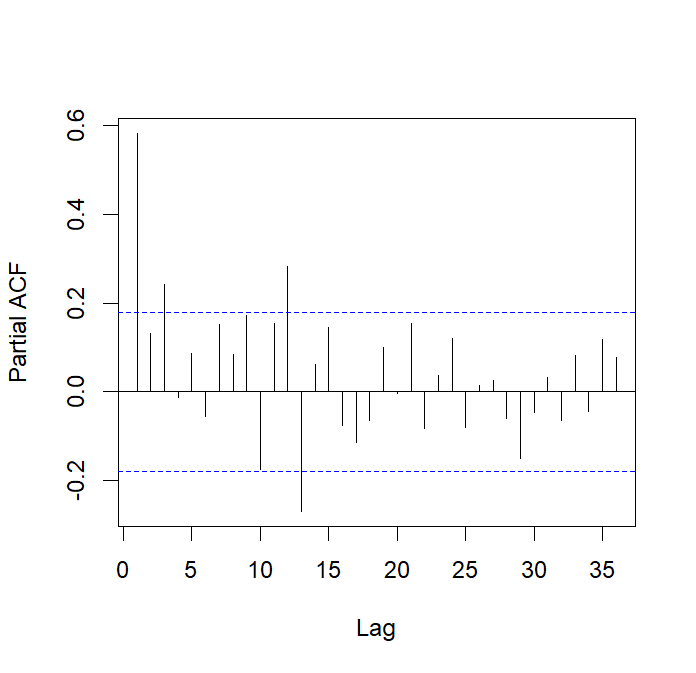 |
| 60–69 | 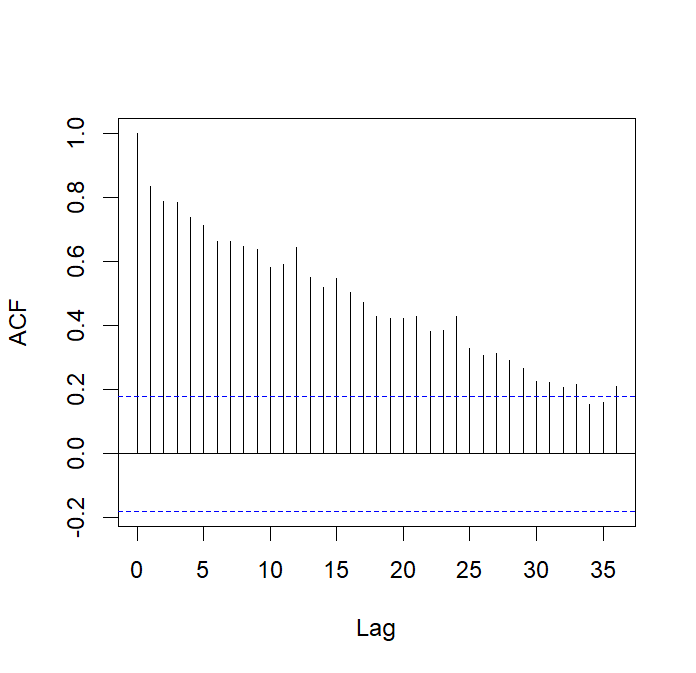 | **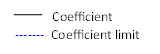**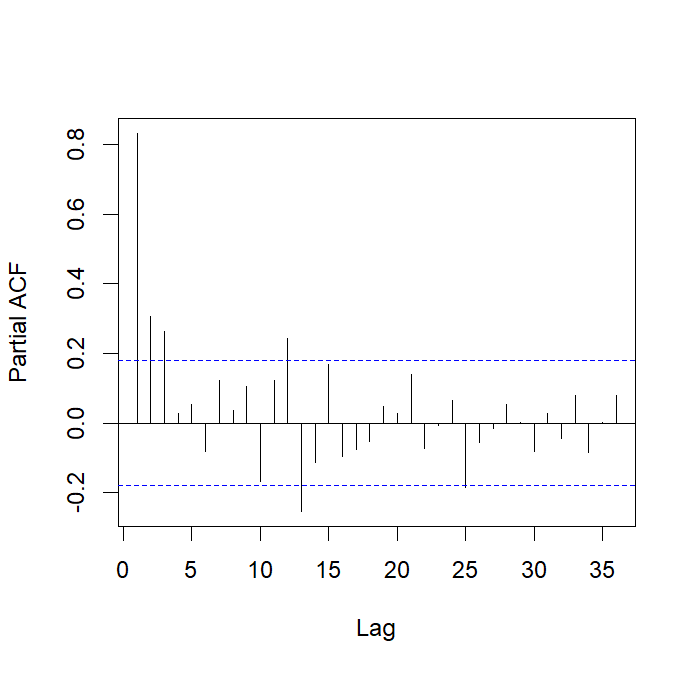 |
| 70–79 | 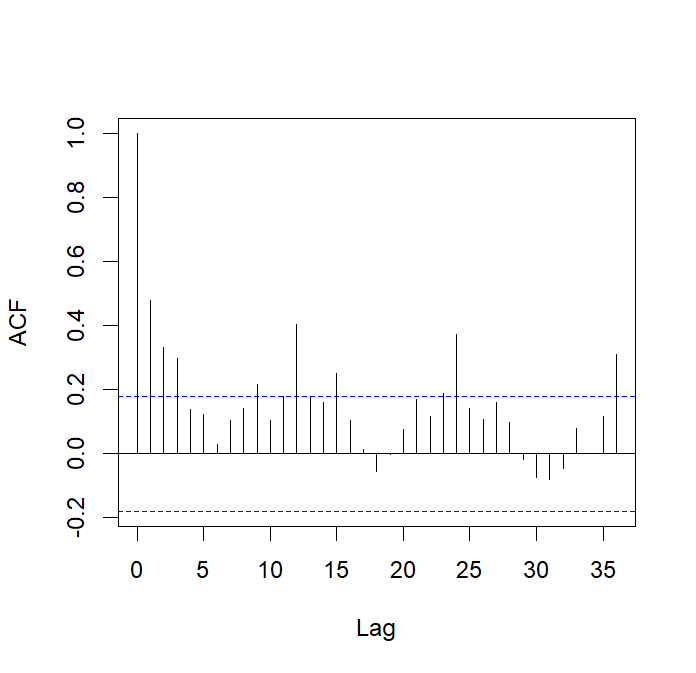 | **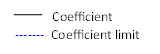**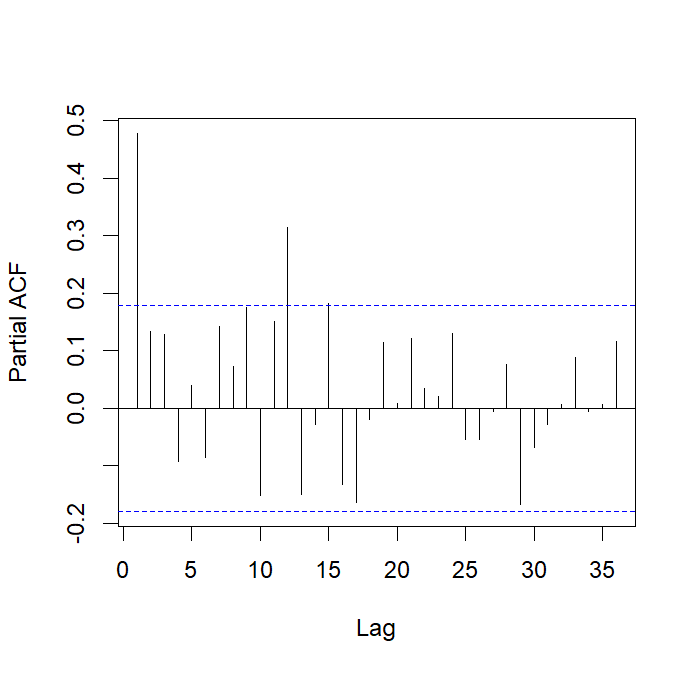 |
| 80+ | 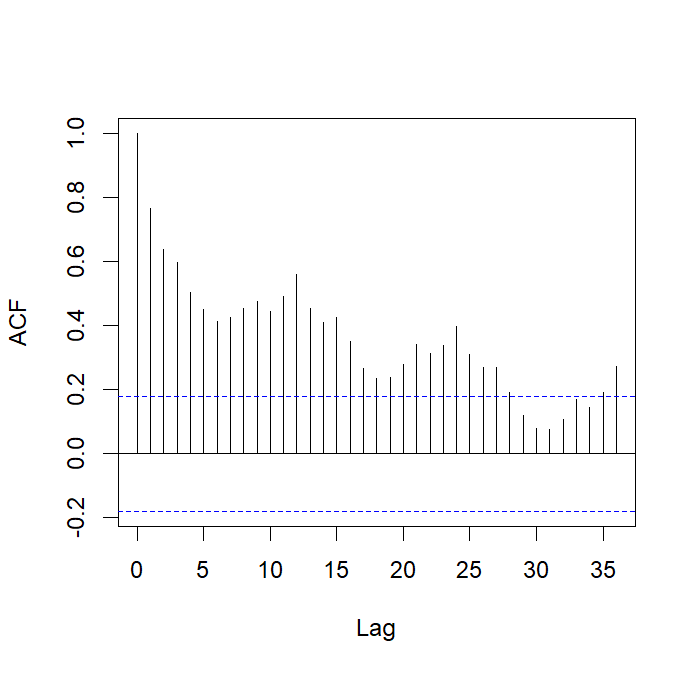 | **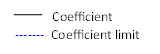**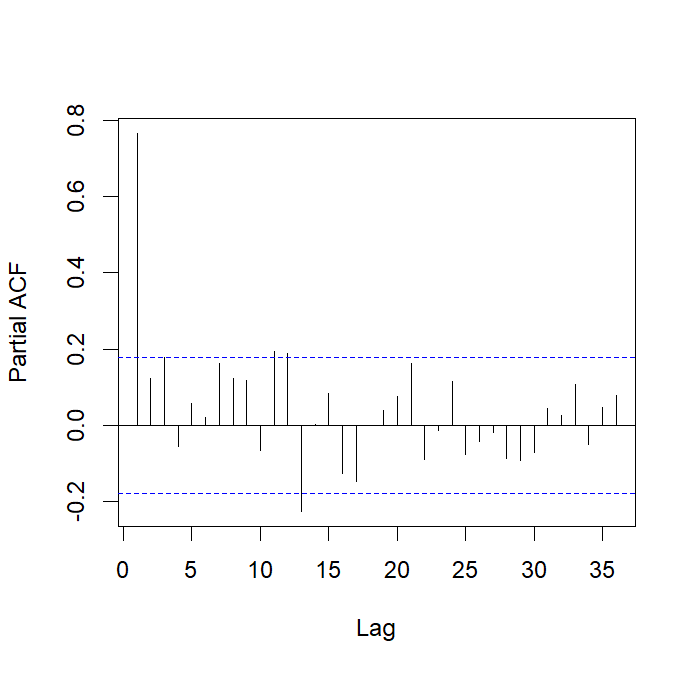 |
| ***Region*** |  |  |
| Bangkok | 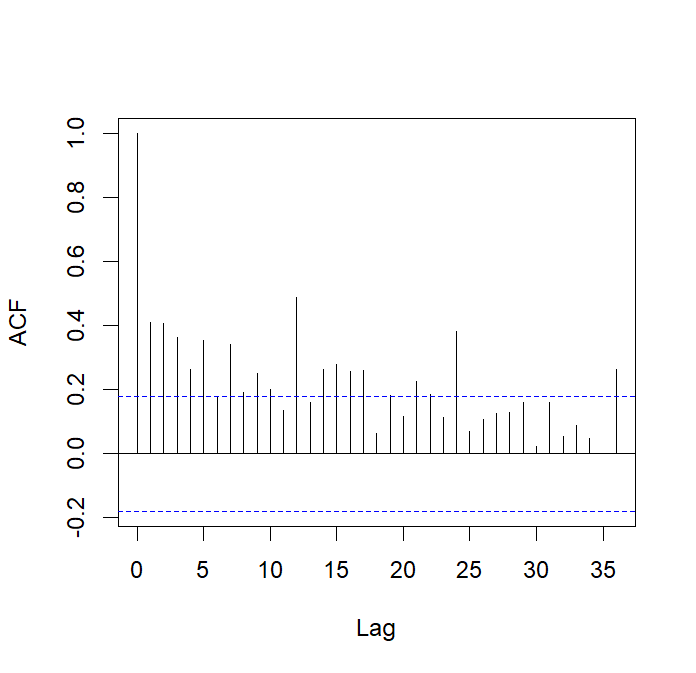 | **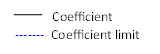**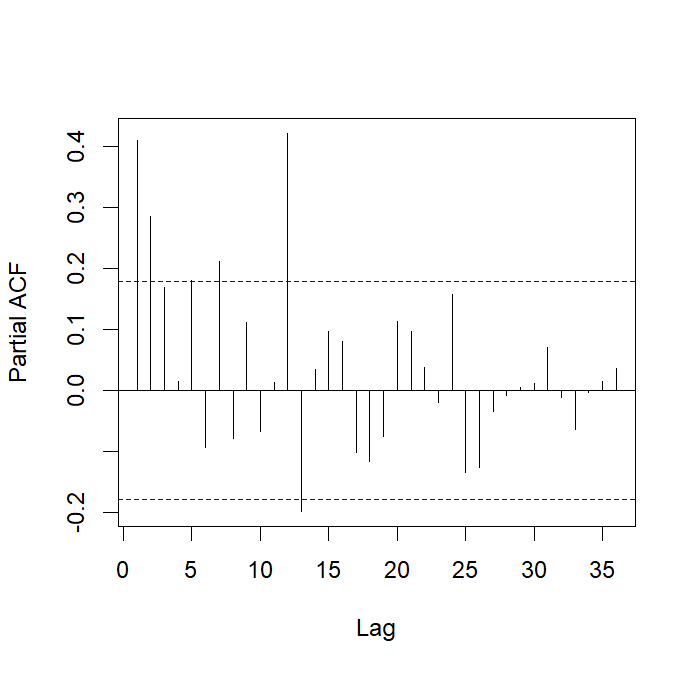 |
| Central | 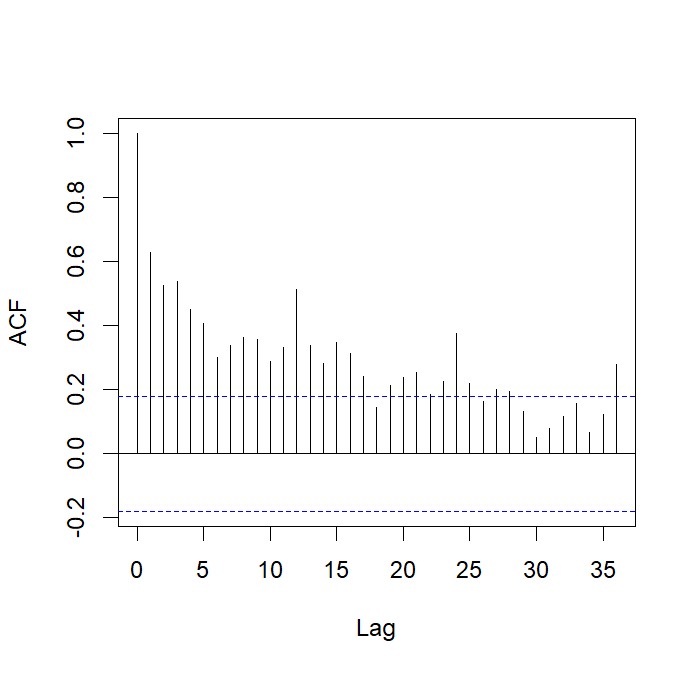 | **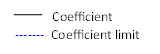**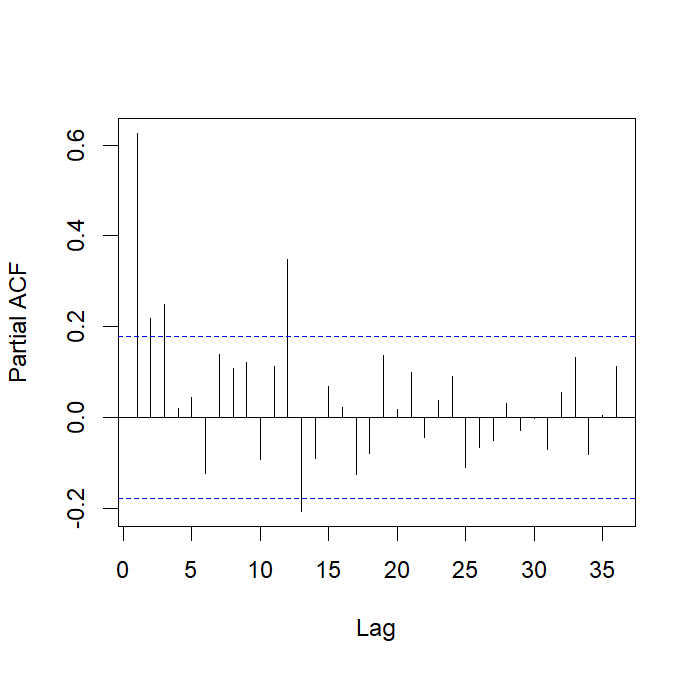 |
| North | 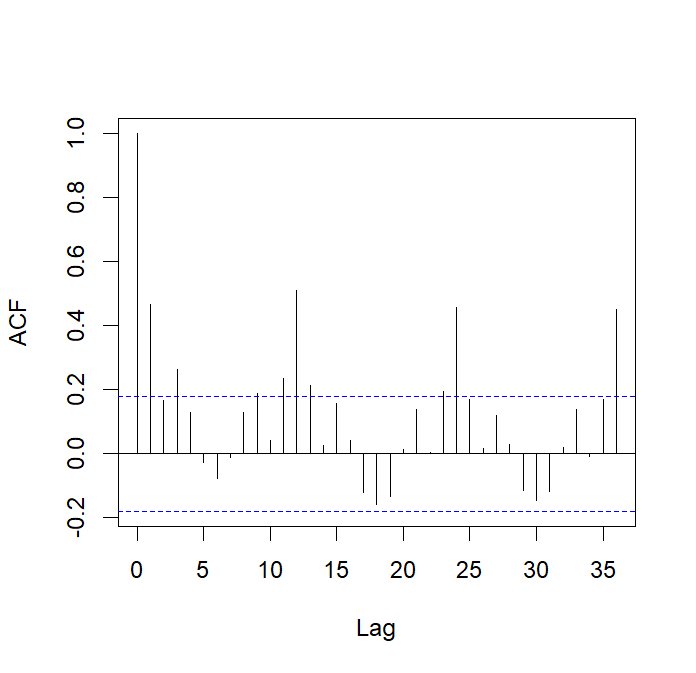 | **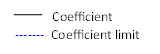**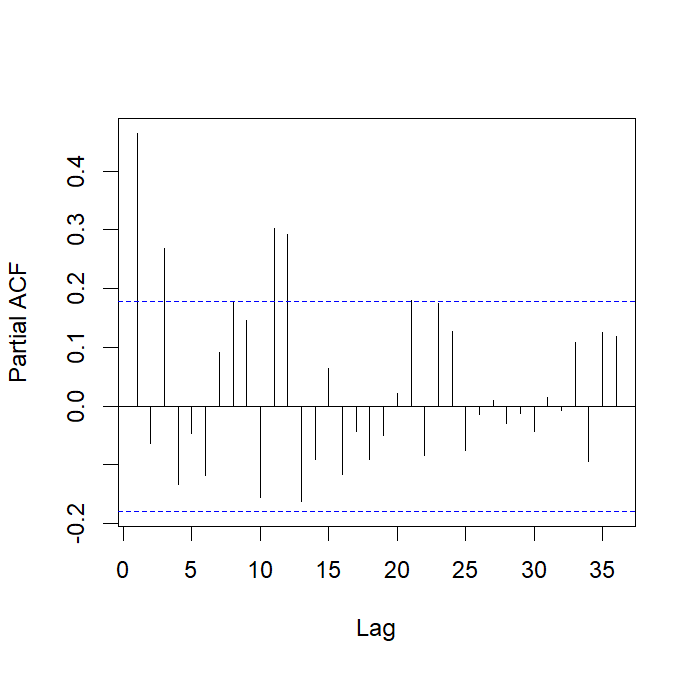 |
| South | 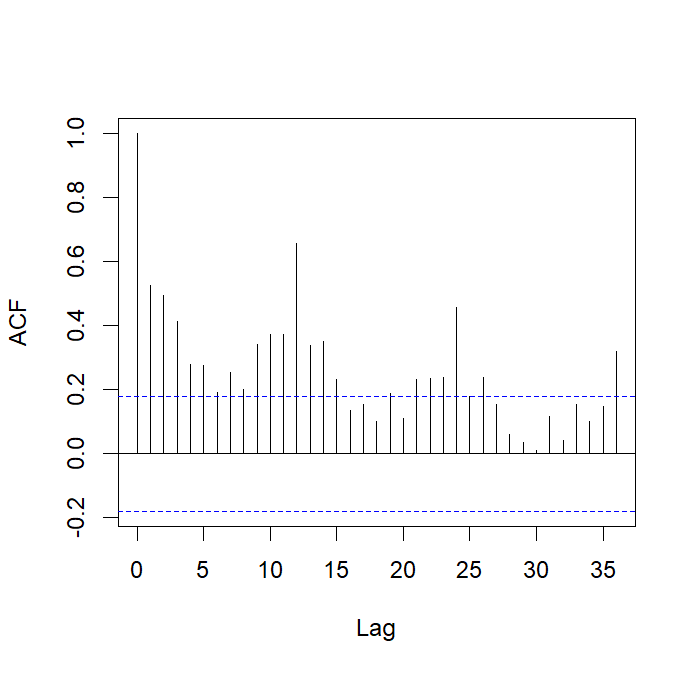 | **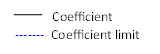**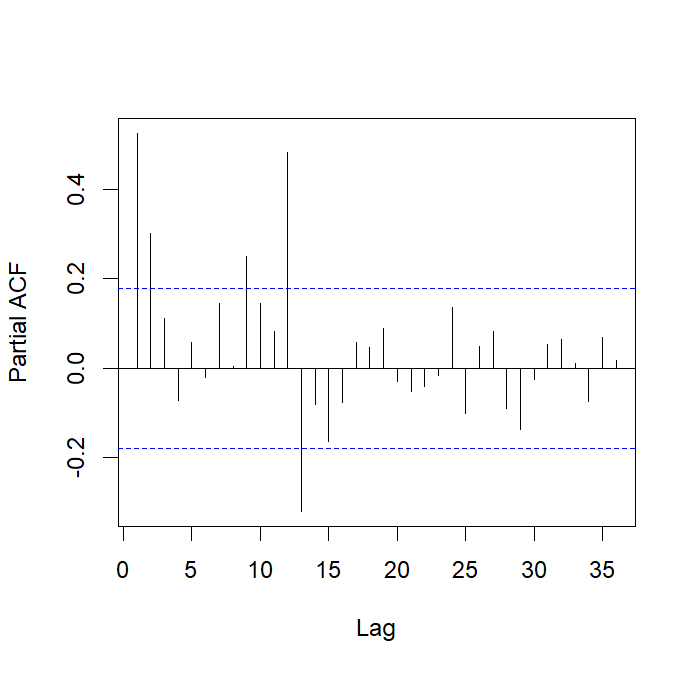 |
| Northeast | 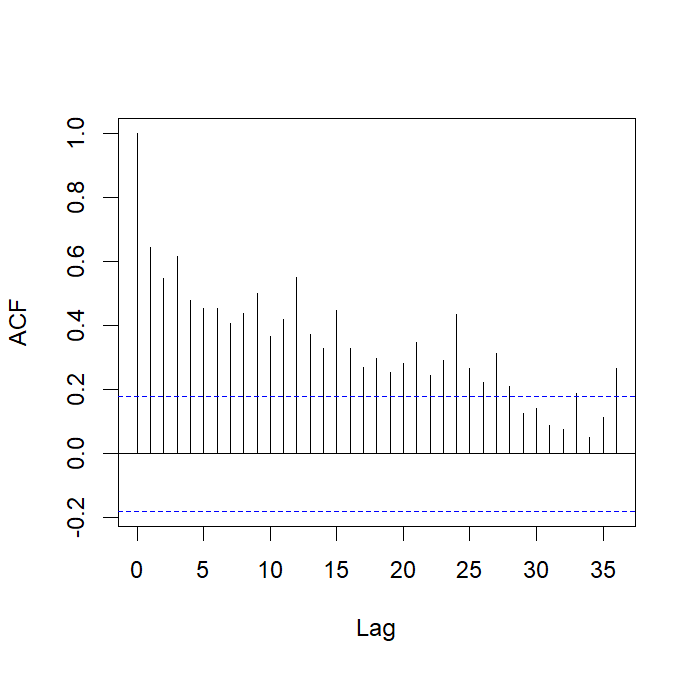 | **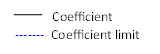**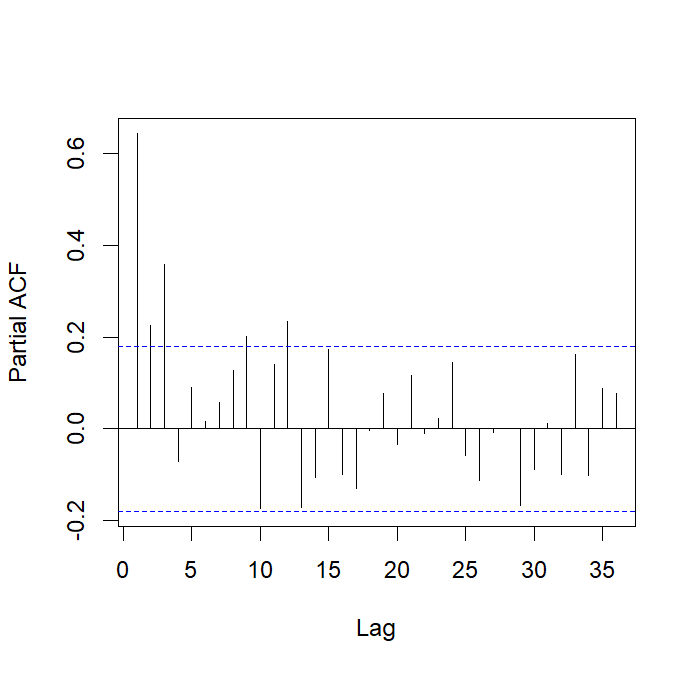 |
| **Women** | | |
| ***Total*** | 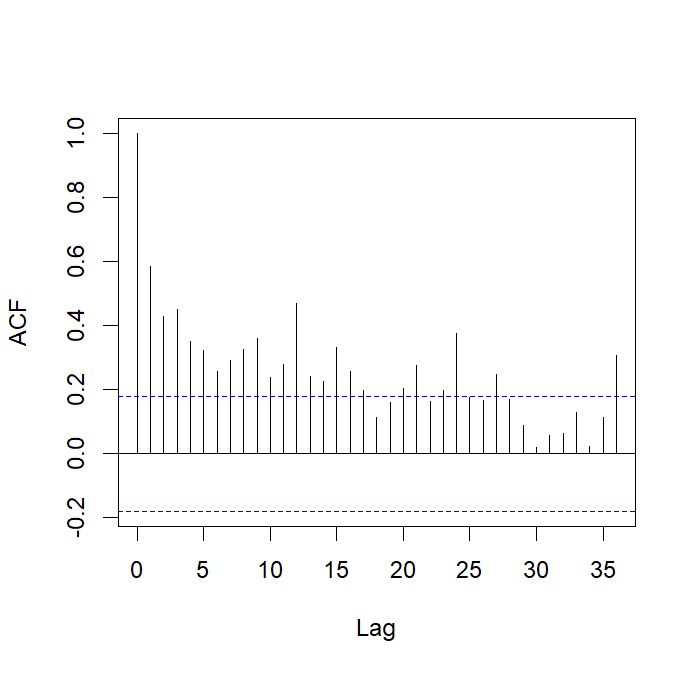 | **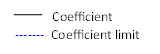**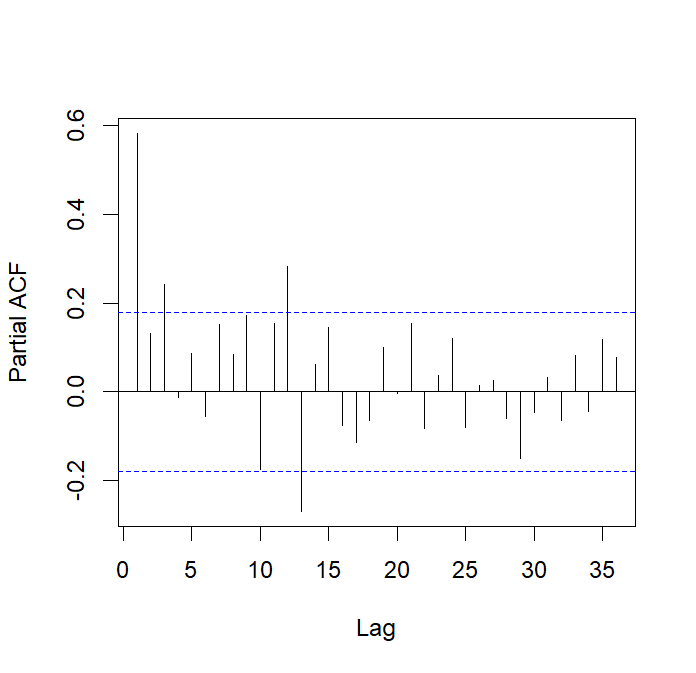 |
| ***Age group*** |  |  |
| 0–14 | 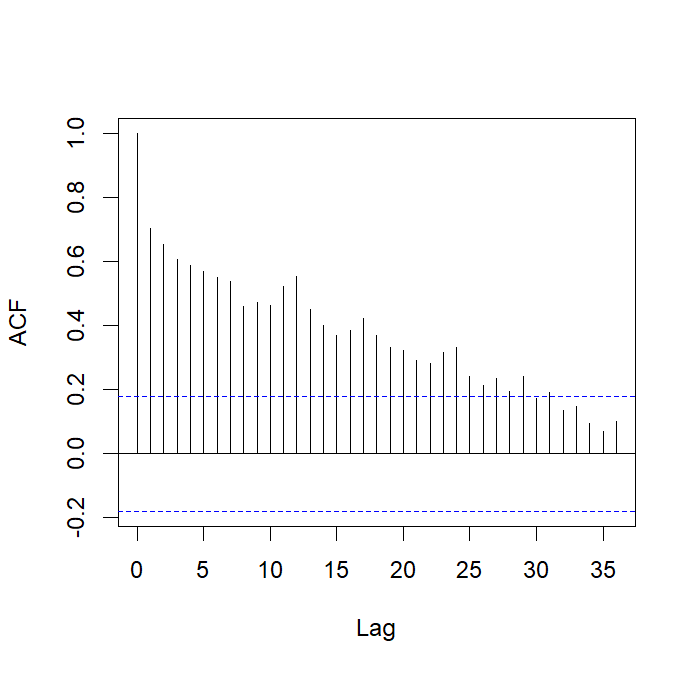 | **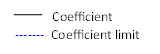**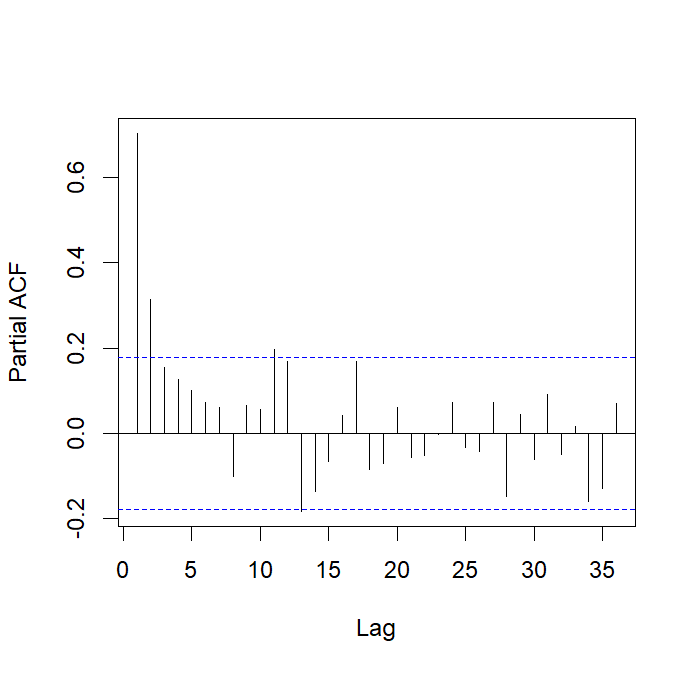 |
| 15–34 | 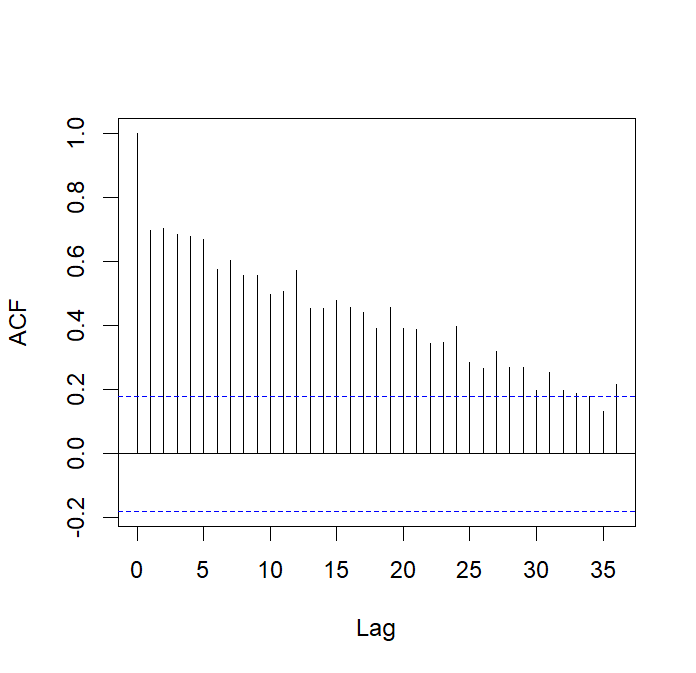 | **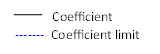**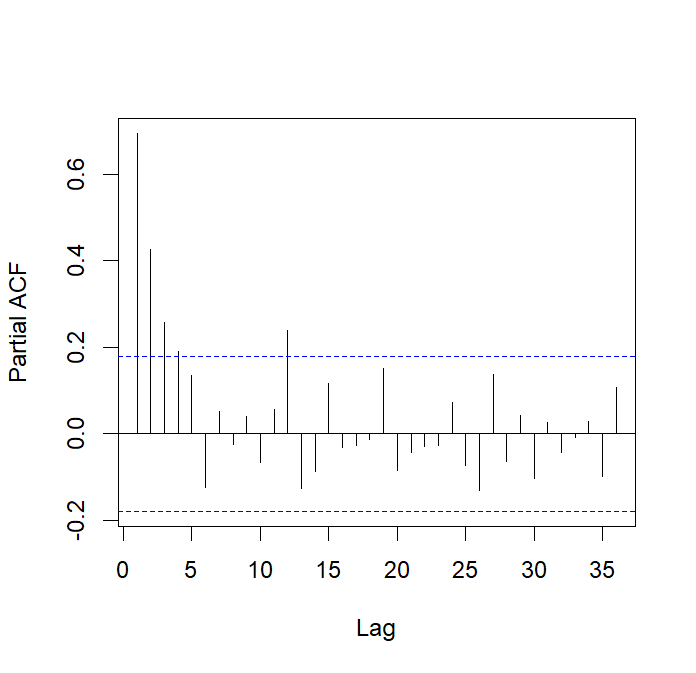 |
| 35–59 | 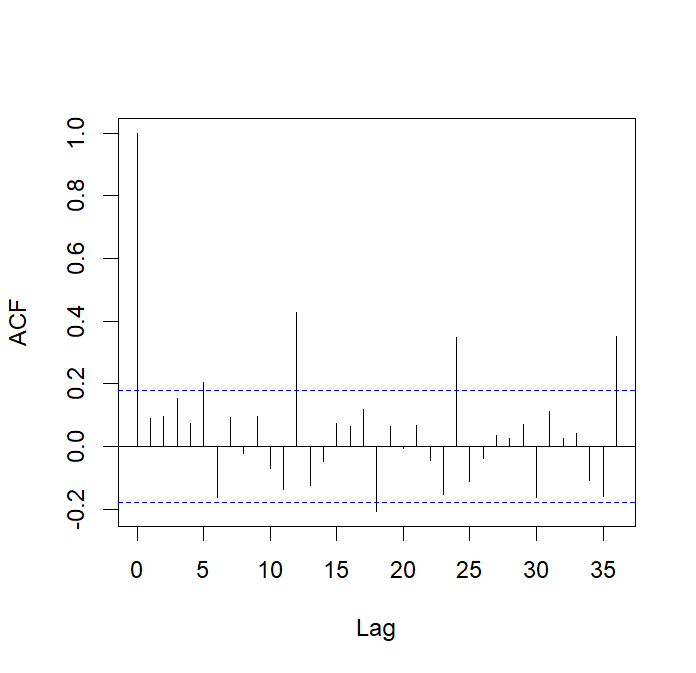 | **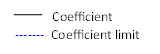**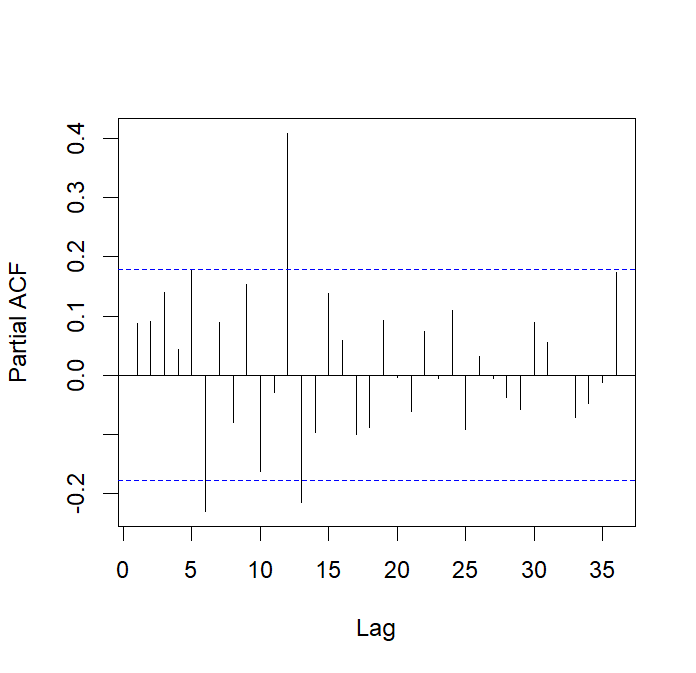 |
| 60–69 | 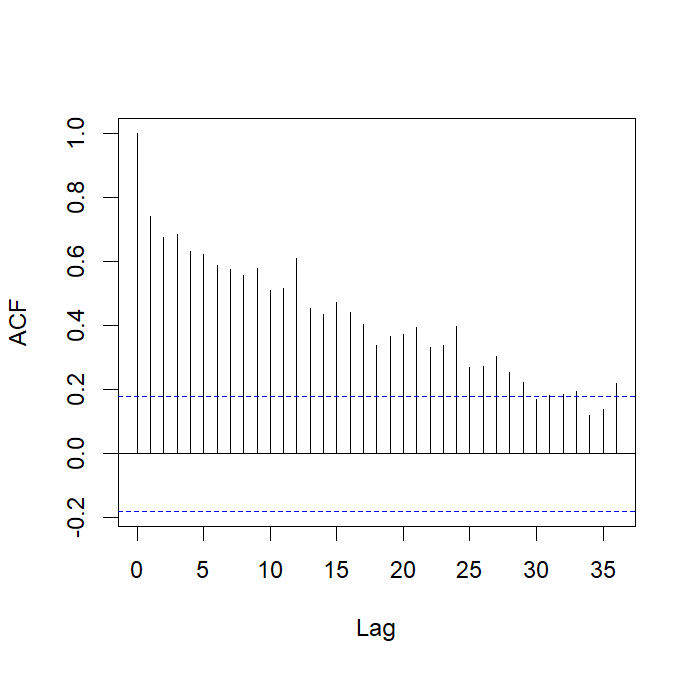 | **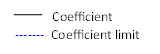**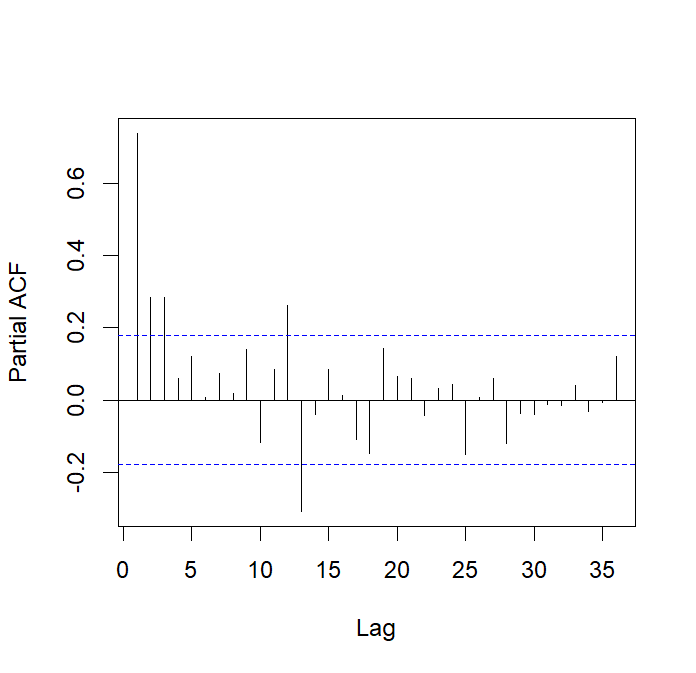 |
| 70–79 | 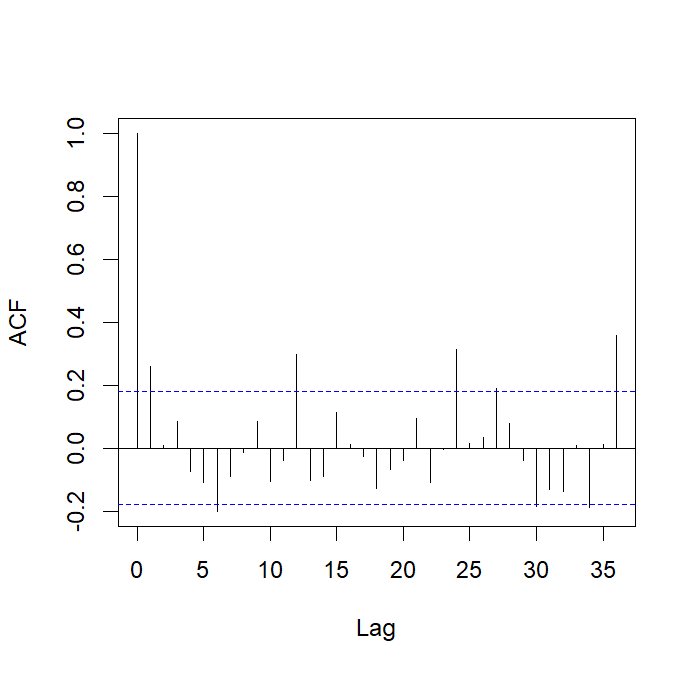 | **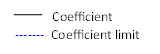**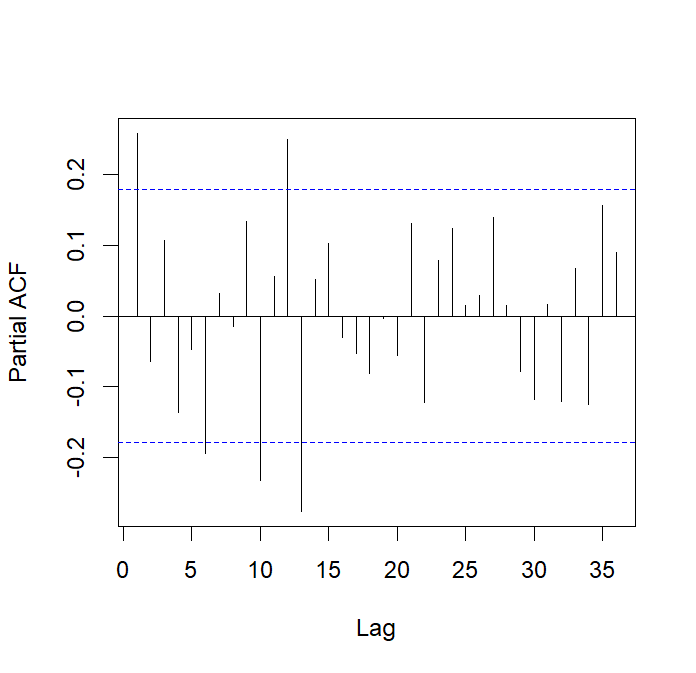 |
| 80+ | 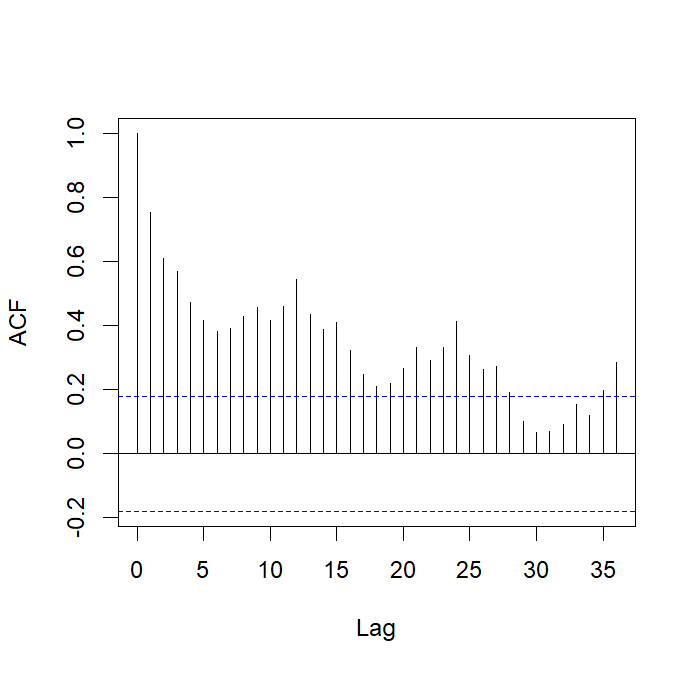 | **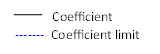**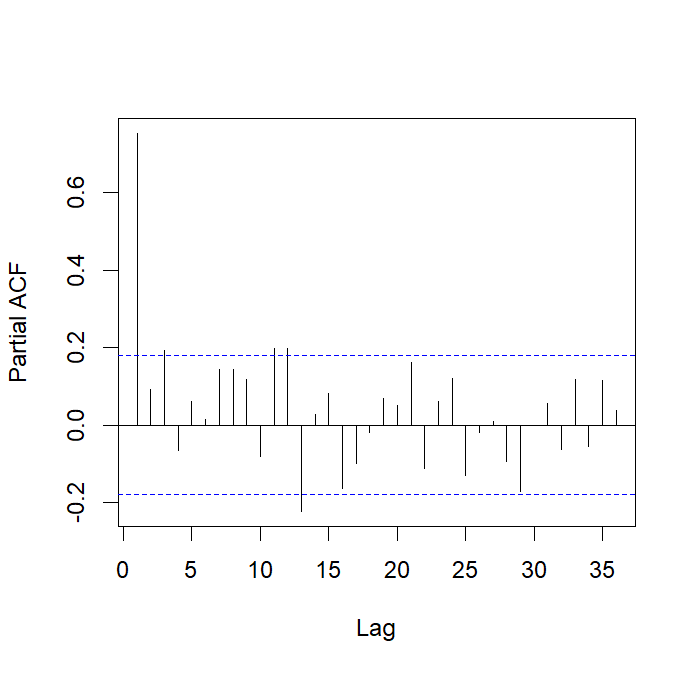 |
| ***Region*** |  |  |
| Bangkok | 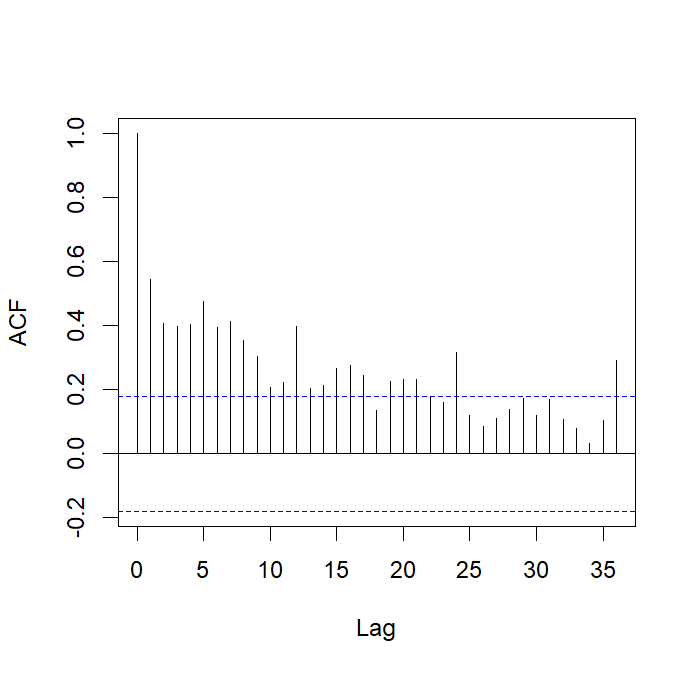 | **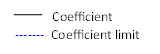**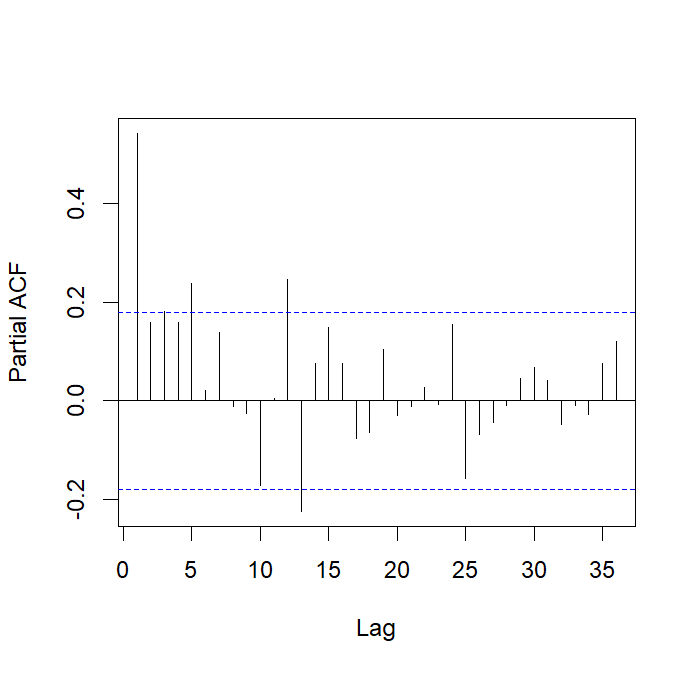 |
| Central | 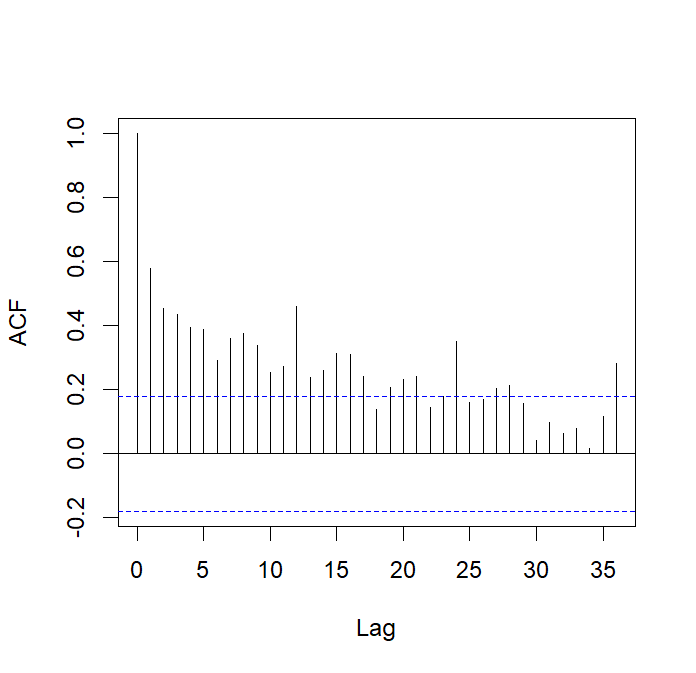 | **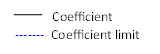**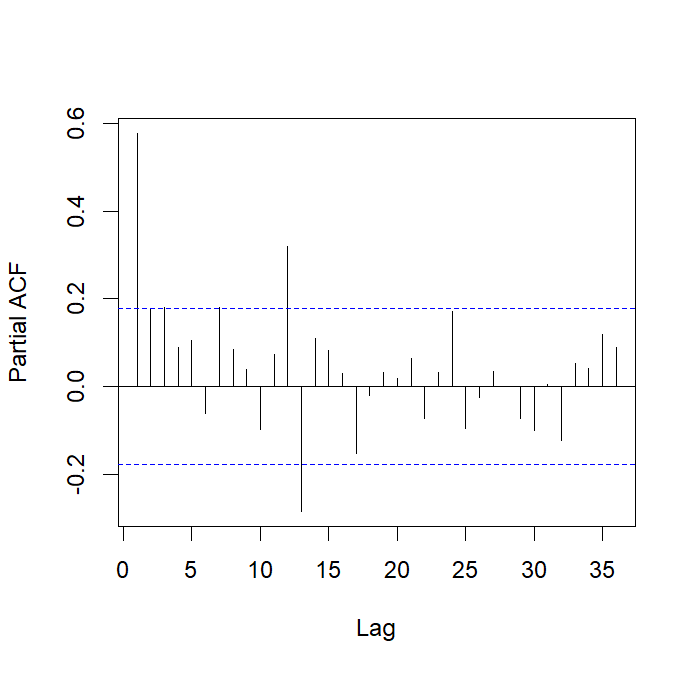 |
| North | 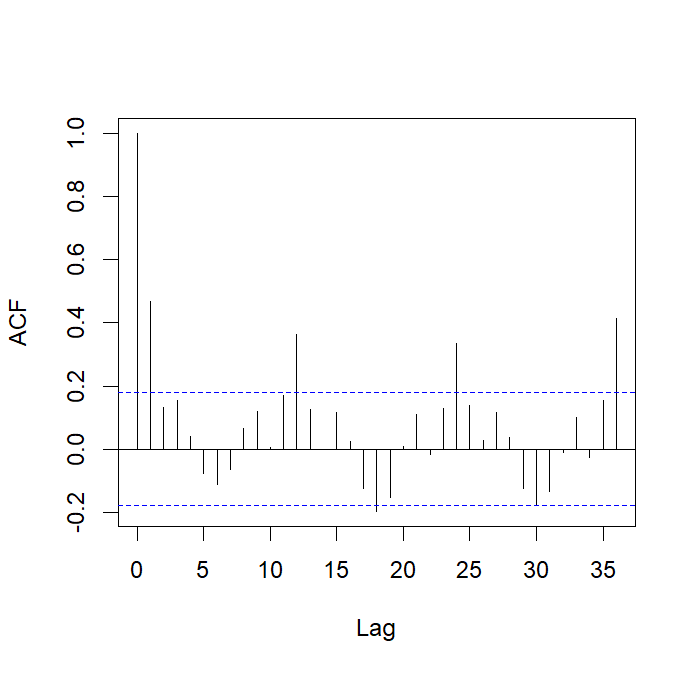 | **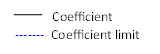**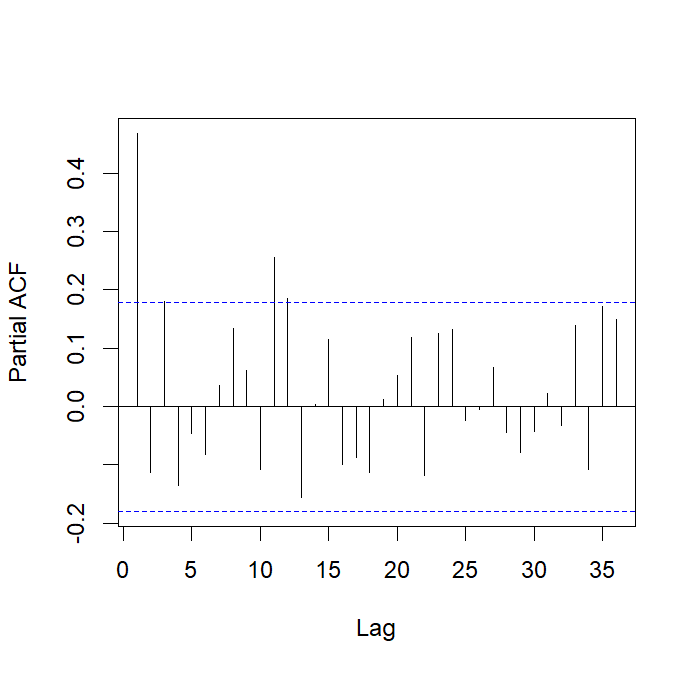 |
| South | 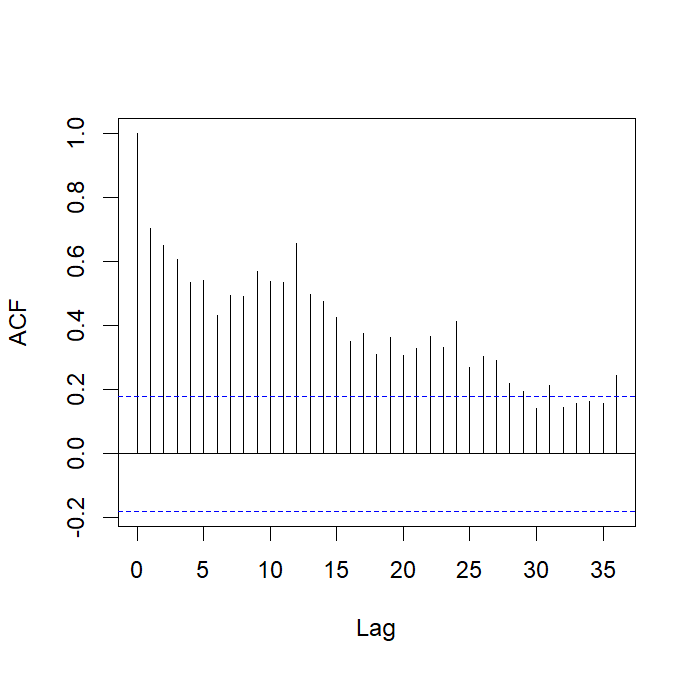 | **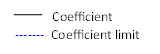**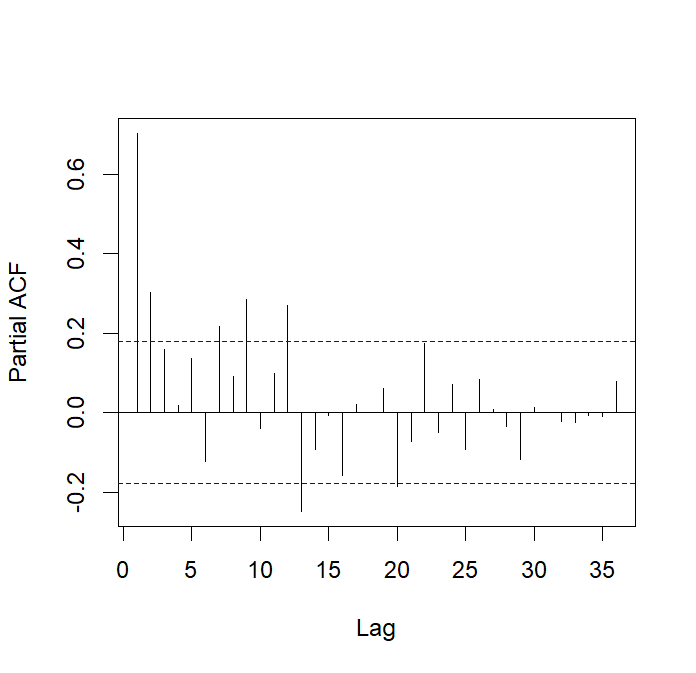 |
| Northeast | 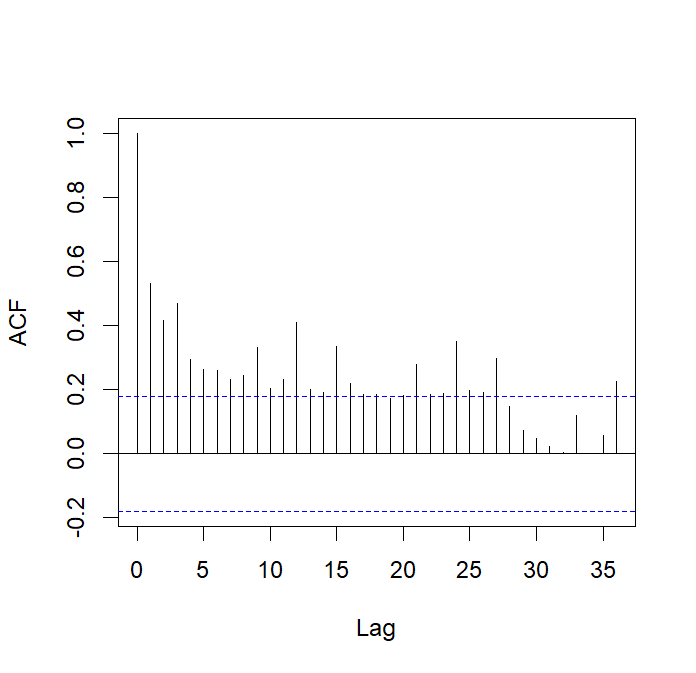 | **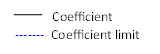**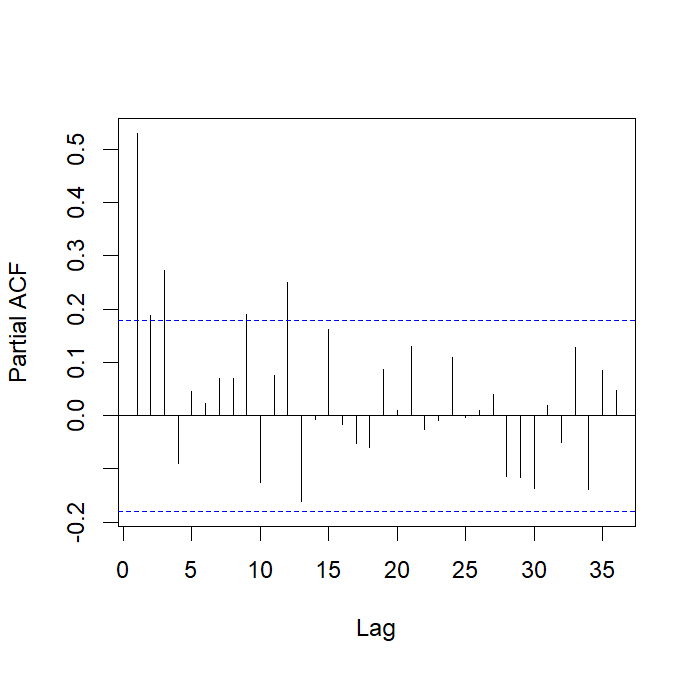 |
